# Boosting Photo‐Pyroelectric Effect via Tunable Polarization and Interfacial Defect Engineering

**DOI:** 10.1002/advs.202519280

**Published:** 2026-02-21

**Authors:** Yanli Huang, Haifen Luo, Jie Yin, Shuai Cao, Gaolei Dong, Zhi Tan, Chunlin Zhao, Peiye Wen, Fengwei Sun, Shan Zhang, Xianzeng Zhang, Yunlu Dai, Zhen Yang, Wei Huang

**Affiliations:** ^1^ Key Laboratory of Opto‐Electronic Science and Technology for Medicine of Ministry of Education Fujian Provincial Key Laboratory of Photonics Technology Fujian Key Laboratory of Flexible Electronics Strait Institute of Flexible Electronics (SIFE Future Technologies) College of Photonic and Electronic Engineering Fujian Normal University Fuzhou China; ^2^ College of Physics Sichuan University Chengdu China; ^3^ Institute of High Performance Computing (IHPC) Agency for Science, Technology and Research (A*STAR) Singapore Singapore; ^4^ College of Materials Science and Engineering Fuzhou University Fuzhou China; ^5^ College of Materials Science and Engineering Sichuan University Chengdu China; ^6^ Faculty of Health Sciences and MoE Frontiers Science Center for Precision Oncology University of Macau Taipa Macau SAR China; ^7^ State Key Laboratory of Flexible Electronics & Institute of Flexible Electronics Northwestern Polytechnical University (NPU) Xi'an China

**Keywords:** barium titanate, interfacial defects, photo‐pyroelectric effect, reactive oxygen species, tumor therapy, tunable polarization

## Abstract

Pyroelectric catalysis has shown promising prospects for sustainable energy generation and medical treatments. However, its potential is limited by intrinsically low pyroelectric coefficients and insufficient interfacial reactivity, resulting in poor reactive oxygen species (ROS) output. In this study, we design Ba(Ti_0.85_Zr_0.15_)O_3_ (BTZ) nanocatalysts, featuring enhanced polarization tunability and oxygen‐vacancy‐rich interfaces, for efficient NIR‐II‐driven photo‐pyroelectric cancer therapy. Molecular dynamics and phase‐field simulations indicate that Zr incorporation maintains strong polarization while facilitating rapid polarization switching via multiscale nanodomain formation. This results in an ultrahigh pyroelectric coefficient (3505 µC m^−2^ K^−1^), representing a 678% enhancement over pristine BaTiO_3_. Interface engineering introduces oxygen vacancies that enhance NIR‐II photothermal conversion and serve as reactive sites to facilitate the dissociation of water molecules. Density functional theory calculations reveal that Zr doping narrows the bandgap and redistributes conduction band electrons, while interfacial oxygen vacancies facilitate water adsorption through optimized hydroxyl binding. As a result, synergistic pyrocatalysis and peroxidase‐like activity under NIR‐II‐driven mild thermal cycling enable robust multipath ROS generation. Both in vitro and in vivo studies confirm efficient tumor cell ablation via NIR‐II induced pyroelectric therapy. This work presents a co‐engineering strategy integrating polarization and interface design to overcome long‐standing limitations in pyroelectric catalysis, advancing its application in precision oncology.

## Graphical abstract

This study reports Ba(Ti_0.85_Zr_0.15_)O_3_ nanocatalysts with enhanced polarization and oxygen‐vacancy‐rich interfaces for efficient NIR‐II photo‐pyroelectric therapy. Zr doping boosts the pyroelectric coefficient and accelerates polarization switching, while oxygen vacancies improve photothermal conversion and surface reactivity. The co‐engineering strategy achieves robust ROS generation, enabling effective tumor ablation and advancing pyroelectric catalysis for precision oncology.

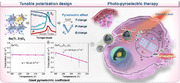

## 1 Introduction

Pyroelectric materials convert thermal fluctuations into electric charges through temperature‐induced changes in polarization, finding broad applications in infrared sensing, motion detection, waste heat recovery, and medical diagnostics [1, 2]. Recently, a new frontier has emerged for ferroelectric materials in electrochemical catalysis driven by the pyroelectric effect (PCE) [3]. This approach leverages the pyroelectric charge generated by ambient temperature variations to drive surface electrochemical reactions, particularly the generation of reactive oxygen species (ROS), such as hydroxyl (•OH), superoxide (•O_2_^−^), and singlet oxygen (^1^O_2_). Potential applications include air purification, water disinfection, and hydrogen generation via water splitting [4, 5]. Particularly, it holds particular promise in pyrodynamic cancer therapy, where ROS production can be harnessed for targeted tumor ablation. However, PCE is constrained by two critical bottlenecks: (i) intrinsically low pyroelectric coefficients (*ρ* < 1,000 µC m^−2^ K^−1^) limit thermal‐to‐electrical energy conversion [3, 6], and (ii) inadequate interfacial catalytic activity due to a lack of active sites for efficient reactant adsorption and dissociation [7, 8]. These constraints hinder ROS generation and limit catalytic effectiveness.

**FIGURE 1:**
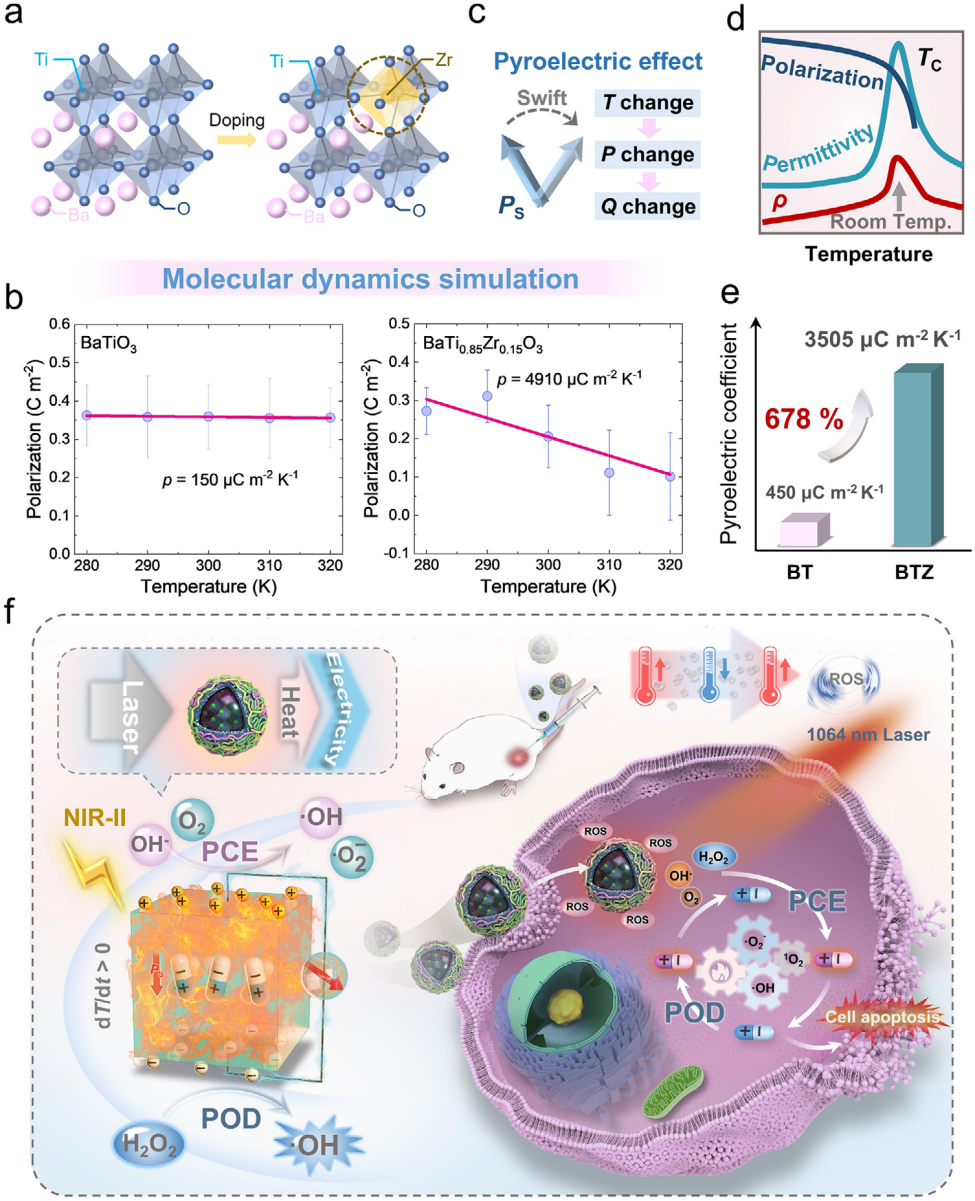
Construction of BTZOX‐P3 NCs for photo‐pyroelectric therapy. (a) Structural design of Zr‐substituted barium titanate. (b) Molecular dynamics simulations of temperature‐dependent polarization in BTO and BTZ. (c) The pyroelectric effect involves polarization change and charge change induced by temperature change. (d) Temperature dependence of polarization, permittivity, and pyroelectric coefficient in modified BTZ. (e) The pyroelectric coefficient obtained from the experiment. (f) Schematic of NIR‐II‐driven pyroelectric therapy of BTZOX‐P3 for tumor elimination.

Perovskite barium titanate (BaTiO_3_, BTO) exhibits large polarization and biocompatibility [9, 10], but exhibits low *ρ* (∼200 µC m^−2^ K^−1^) due to stable ferroelectric domains, which suppresses the temperature derivative of polarization (∂*P*/∂*T*, *P* is polarization) [3]. Isovalent ion substitution destabilizes ferroelectric phases by introducing atomic‐scale lattice distortions, breaking long‐range ferroelectric order to form nanoscale domains or polar nanoregions (<10 nm) that facilitate polarization switching under thermal fluctuations [11, 12, 13]. An effective strategy must concurrently shift the Curie temperature (*T*_C_) toward room temperature while preserving an ordered ferroelectric phase with moderate domain size and a sharp phase transition to retain high polarization and ∂*P*/∂*T*. Zirconium (Zr) ions substitution for the Ti‐site provides sufficient lattice strain for *T_C_* tuning without destabilizing the perovskite structure (Figure 1a). Molecular dynamics simulations confirm that 15% Zr‐doped in Ba(Ti_0.85_Zr_0.15_)O_3_ exhibits rapid polarization changes within 280–320 K, yielding a giant pyroelectric coefficient of 4910 µC m^−2^ K^−1^, markedly higher than 150 µC m^−2^ K^−1^ observed in pristine BaTiO_3_ (Figure 1b). This approach is expected to preserve large polarization while sustaining a giant pyroelectric effect near physiological temperature.

Alongside the enhanced intrinsic pyroelectric coefficients, the efficiency of PCE also hinges on the magnitude and rate of temperature variation. Second near‐infrared (NIR‐II) window light serves as an ideal trigger, offering deep tissue penetration and precise spatiotemporal thermal cycling [14, 15, 16, 17]. While previous approaches have incorporated external photothermal agents, such as nanoheterojunction (e.g., BT@Nb, CeO_2–_BT‐IR1061, BT‐CuS@PDA) [14, 15, 18], plasmonic hybrids (BT@Au) [5, 19], or dopamine coatings [20, 21], to improve heat conversion, these often complicate biocompatibility and scalability. Currently, most pyroelectric compounds can only achieve photothermal performance under NIR‐I stimulation, and realizing efficient activation under NIR‐II remains a significant challenge due to the unique optical and thermal properties required. Alternatively, interface engineering via oxygen vacancy formation offers a unified solution: oxygen defects not only extend NIR absorption, but also introduce unsaturated sites that promote dissociative adsorption of H_2_O and O_2_, thereby facilitating •O_2_^−^ and •OH generation. This interface‐level activation enables pyroelectrics to retain the advantages of catalytic responsiveness while overcoming their inherent limitations in light absorption and surface reactivity.

Guided by molecular dynamics simulations, we developed Ba(Ti_0.85_Zr_0.15_)O_3_ nanocatalysts (BTZ) engineered with interfacial oxygen vacancies for photo‐pyroelectric tumor therapy. Zr substitution lowered *T*_C_ to near‐physiological levels and induced convergent phase transitions, enabling a giant dielectric constant and rapid temperature‐dependent polarization shifts (Figure 1c,d). This yields an ultrahigh pyroelectric coefficient (3505 µC m^−2^ K^−1^), representing a 678% enhancement over pristine BaTiO_3_ (Figure 1e). Furthermore, interface engineering introduced oxygen vacancies (BTZOX), enhancing NIR‐II‐induced photothermal conversion and providing active sites for dissociative H_2_O adsorption. Under NIR‐II‐induced thermal cycling, BTZOX generated substantial ROS through synergistic pyrocatalytic and peroxidase (POD)‐like activity. Density functional theory (DFT) calculations revealed that Zr doping narrowed the bandgap, redistributed conduction band electrons, and interface oxygen vacancy sites activated H_2_O adsorption via anisotropic charge redistribution, synergistically facilitating a multipath ROS storm. A layer‐by‐layer self‐assembly of polyethyleneimine (PEI), polyacrylic acid (PAA), and polyethylene glycol (PEG) formed P3 coating, endowing BTZOX (BTZOX‐P3) with enhanced biocompatibility. Both in vitro and in vivo studies confirmed effective tumor ablation of BTZOX‐P3 nanocatalysts (NCs) via ROS bursts during mild thermal cycling (35°C–45°C; Figure 1f). This work presents a co‐engineering strategy of polarization and interface to boost PCE performance, advancing a scalable and biocompatible platform for NIR‐II‐triggered precision oncology.

## 2 Results and Discussion

### 2.1 Design and Characterization of BTZOX‐P3 Nanocatalysts

**FIGURE 2:**
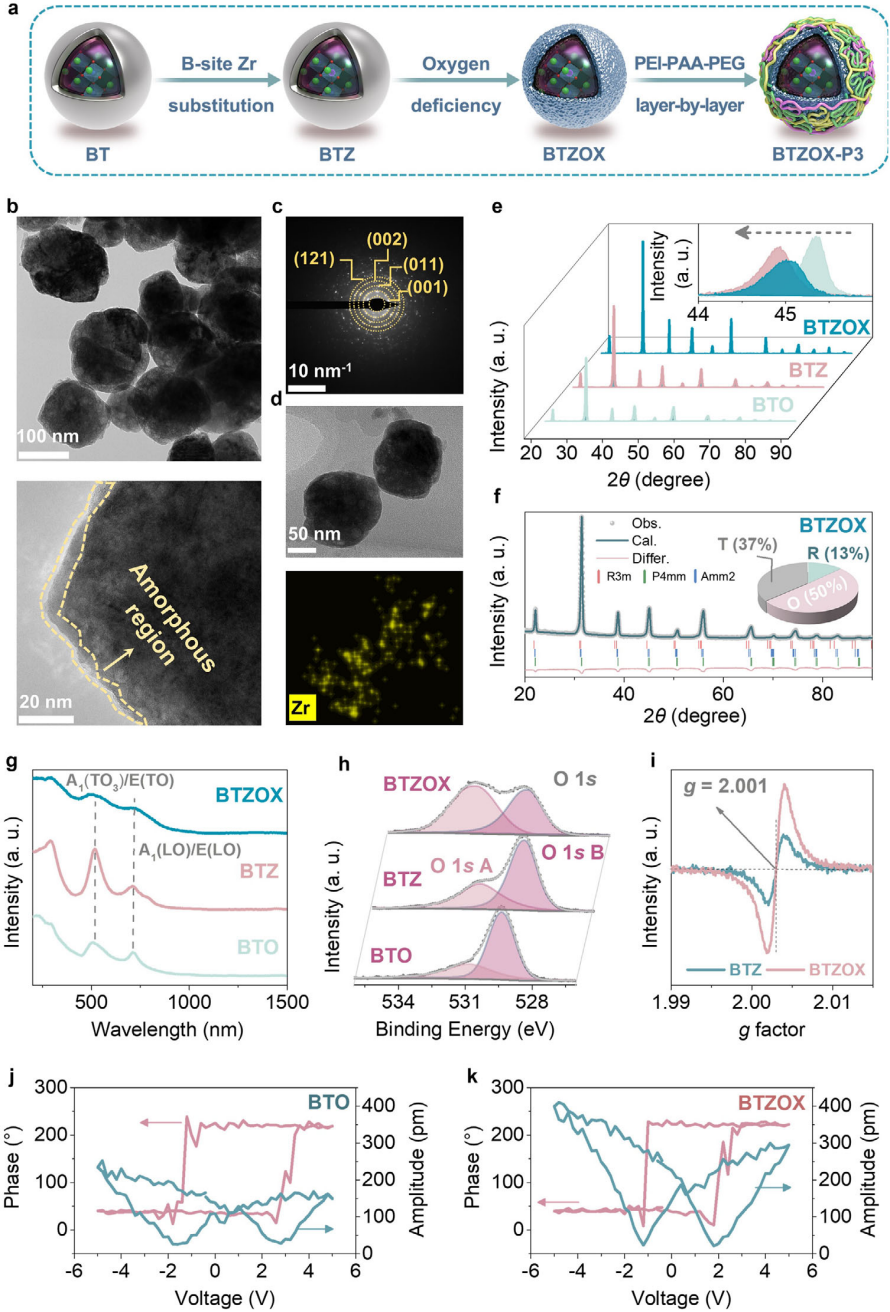
Design and structure characterization of BTZOX‐P3 NCs. (a) Preparation process for BTZOX‐P3 NCs. (b) TEM image of BTZOX‐P3. (c) SAED pattern and (d) element mapping of BTZOX‐P3. (e) XRD patterns of BT, BTZ, and BTZOX. (f) Rietveld refinement of BTZOX. (g) Raman spectra. (h) XPS spectra of O1*s*. (i) EPR spectra of BTZ and BTZOX. PFM characterization of (j) BTO and (k) BTZOX nanoparticles, showing ferroelectric phase hysteresis and amplitude loops.

The synthesis of BTZOX‐P3 NCs was illustrated in Figure 2a. BTZOX NCs were fabricated via the hydrothermal method and treated with sodium borohydride. To improve colloidal stability and biocompatibility, a tri‐layer polymer coating was assembled on the BTZOX surface. First, cationic PEI was coated onto the nanoparticles, followed by the modification of a pre‐synthesized PAA‐PEG copolymer. This electrostatic self‐assembly process was monitored by the Zeta potential and dynamic light scattering method (Figure S1). As shown in Figure S1a, the negative potential of pristine BTZOX (−27 mV) reversed to +28 mV after PEI coating due to protonated amino groups. Subsequently, the introduction of the anionic PAA‐PEG copolymer restored a negative potential (−22.8 mV) through the electrostatic interaction between the carboxyl groups of PAA and the amine groups of PEI. Correspondingly, the hydrodynamic diameter (Figure S1b) increased from ∼155 to ∼290 nm after PEI coating, and finally stabilized at ∼220 nm for the BTZOX‐P3 NCs. Furthermore, the colloidal stability was evaluated by monitoring the size and surface charge variations in PBS over time. As depicted in Figure S1c,d, no significant aggregation or potential shift was observed after 7 days of incubation, demonstrating that P3 coating endowed the nanocatalysts with excellent physiological stability suitable for biomedical applications.

Transmission electron microscope (TEM) images revealed irregularly shaped spherical particles with diameters averaging 140 nm (Figure 2b). A thin amorphous shell, associated with oxygen vacancies and local lattice distortion [22], was clearly visible. Selected‐area electron diffraction (SAED) patterns exhibited concentric rings indexed to the (001), (011), (002), and (121) crystal planes, confirming the polycrystalline structure of BTO (Figure 2c). Elemental mapping demonstrated uniform distribution of Ba, Ti, and Zr throughout BTZOX NCs (Figure 2d; Figure S2). X‐ray diffraction (XRD) patterns of BTO, BTZ, and BTZOX showed a pure perovskite structure (Figure 2e), with a shift in the (002) peak indicating unit cell expansion due to the larger Zr^4+^ replacing Ti^4+^. Rietveld refinement of BTZ's phase structure (Figure 2f) identified rhombohedral (R), orthorhombic (O), and tetragonal (T) phases, with content values of 13%, 37%, and 50%, respectively, demonstrating multiphase coexistence in BTZ. The refinement result showed reliable fitting, with *R*_wp_ and goodness‐of‐fit values of 2.72% and 1.28%, respectively (Table S1).

Raman spectroscopy was employed to probe lattice disorder (Figure 2g). In contrast to pristine BTO and BTZ, which displayed sharp and well‐defined peaks, BTZOX exhibited broadened and attenuated signals, indicative of surface disorder induced by oxygen deficiency [23]. To further examine defect chemistry, X‐ray photoelectron spectroscopy (XPS) and electron paramagnetic resonance (EPR) analyses were performed. XPS spectra confirmed the presence of Ba, O, Ti, and Zr (Figure S3a). The Ti 2*p* doublet displayed binding energies of 458 and 464 eV in BTZOX, consistent with Ti^4+^ oxidation states (Figure S3b) [24]. Moreover, discernible Ti^3+^ signals were detected in BTZOX. The O 1*s* profile (Figure 2h) featured two peaks: a main peak at 529 eV (O 1*s* B) and a higher energy satellite peak at ∼532 eV (O 1*s* A), corresponding to the lattice O and absorbed water as well as the appearance of oxygen vacancies and hydroxyl, respectively [24]. Compared to BTZ, the intensity of O 1*s* A increased in BTZOX, signifying an elevation in surface oxygen vacancies and the appearance of the 2Ti^3+^‐$V_{O}^{\cdot \cdot }$ defect clusters [24]. In Ti^4+^‐Ti^3+^ systems, oxygen vacancies tend to capture an electron, resulting in the formation of single‐electron‐trapped oxygen vacancies ($V_{O}^{\cdot }$) with a *g*‐factor of 1.999 [25], as observed in EPR spectra (Figure 2i). Furthermore, the Zr 3*d* spectrum in BTZ and BTZOX displayed a doublet (Zr 3*d*_5/2_ and Zr 3*d*_3/2_), with a spin‐orbit splitting of 2.3 eV [26], and the Zr 3*d*_5/2_ peak at ∼181 eV confirmed the presence of Zr^4+^ (Figure S3c). Furthermore, to validate the ferroelectric functionality at the single‐particle level, piezoresponse force microscopy (PFM) was conducted on the nanoparticles. As displayed in Figure 2j,k, BTO and BTZOX nanoparticles exhibited characteristic butterfly‐shaped amplitude loops and distinct 180° phase hysteresis loops. This direct observation confirmed that the synthesized nanoparticles possessed ferroelectric properties at the nanoscale, ensuring their capability for subsequent pyroelectric catalysis.

### 2.2 Enhanced Pyroelectricity of BTZ System

**FIGURE 3:**
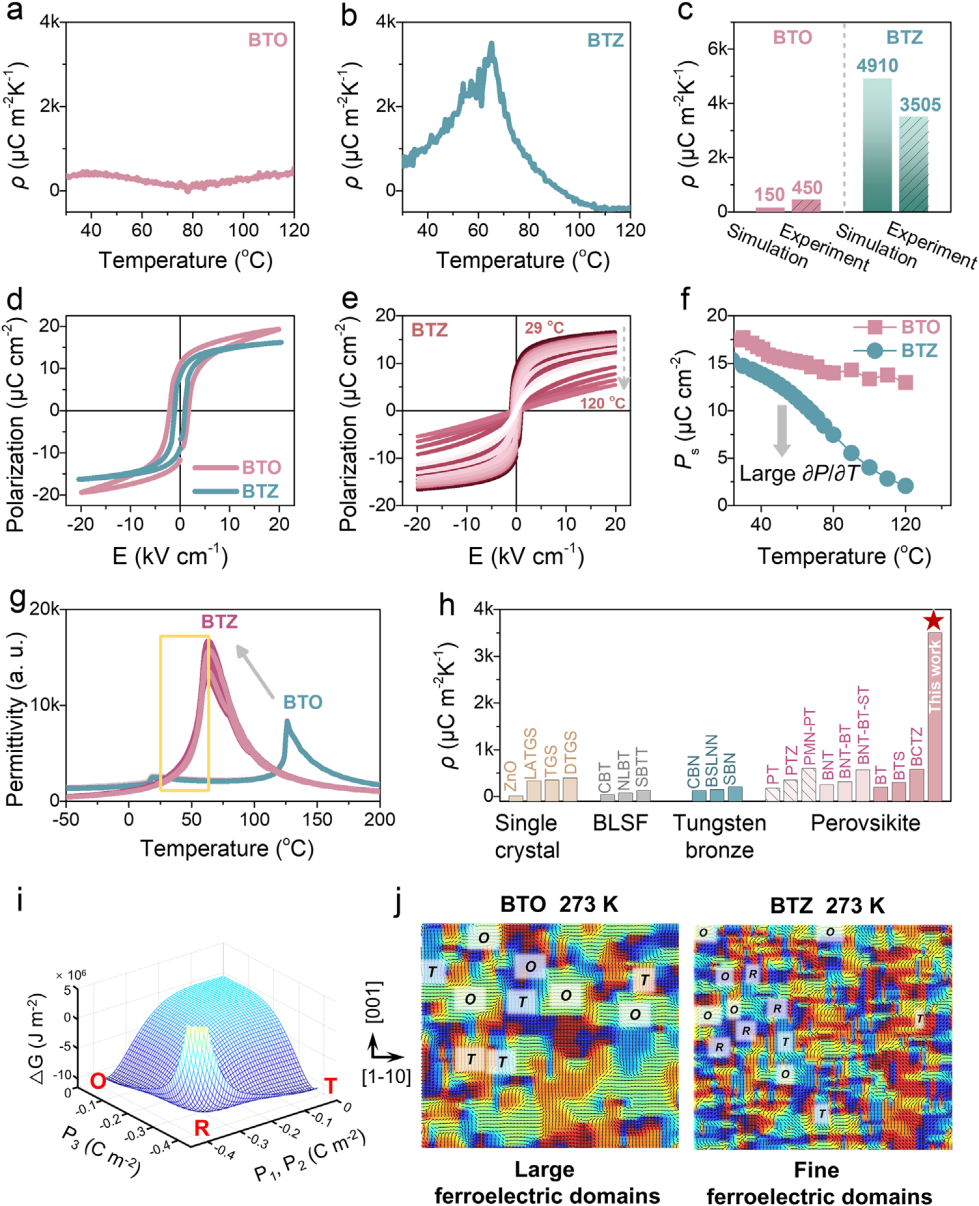
Enhanced pyroelectricity in the BTZ system. The temperature dependence of the pyroelectric coefficient of (a) BTO and (b) BTZ. (c) The pyroelectric coefficient of BTO and BTZ by molecular dynamics simulation and experiment. (d) *P‐E* loops of BTO and BTZ. (e) *P‐E* loops of BTZ measured at different temperatures. (f) *P*_S_ ‐T curves of BTO and BTZ. (g) Temperature‐dependent dielectric constant of BTO and BTZ. (h) The comparison of the pyroelectric coefficient between different pyroelectric materials [28, 29, 30, 31, 32, 33, 34]. (i) 3D free energy barrier along the polarization rotation path of BTZ (*P*_1_ = *P*_2_ = 0, *P*_3_ ≠ 0 for T phase, *P*_1_ = *P*_2_ ≠ 0, *P*_3_ = 0 for O phase, *P*_1_ = *P*_2_ = *P*_3_ ≠ 0 for R phase). (j) 2D domain structures of BTO and BTZ, and their phase structures determination at 273 K.

Following the structural design and characterization of BTZOX‐P3 NCs, the pyroelectric properties of the BTZ system were further investigated using a dedicated pyroelectric coefficient test system. The pyroelectric coefficient *ρ* was defined as [27]:

(1) $$\rho^{\sigma ,\;E}={\left(\frac{dP_{S}}{dT}\right)}_{\sigma ,\;E}$$

where *P*_S_ was the spontaneous polarization, *T* was the temperature, and *σ* and *E* denoted constant stress and electric field conditions. To evaluate the macroscopic electrical properties of BTO and BTZ compositions, the synthesized nanoparticles were pressed and sintered into dense ceramics for characterization. As shown in Figure 3a–c, BTZ exhibited a maximum *ρ* of 3505 µC m^−2^ K^−1^, nearly an eightfold increase compared to the undoped counterpart (450 µC m^−2^ K^−1^), confirming the effectiveness of Zr doping. The experimental coefficient was lower than the MD simulated value (4910 µC m^−2^ K^−1^), because the MD simulation modeled an ideal single‐crystal, whereas the experimental sample was a polycrystalline ceramic where random grain orientation and grain boundaries naturally reduced the macroscopic response. Nevertheless, the experimental result represented a giant enhancement over pristine BTO, validating the effectiveness of the design. Furthermore, according to Equation (1), the enhanced pyroelectric performance via doping arised from increased spontaneous polarization and the resulting change in polarization per unit temperature variation (∂*P*/∂*T*). Polarization‐electric field (*P‐E*) loops (Figure 3d) revealed that doped samples exhibited reduced remanent polarization and coercive field, suggesting that Zr doping weakened ferroelectric ordering and lowered the energy barrier for polarization reversal. Temperature‐dependent *P‐E* loops for BTO (Figure S4) and BTZ (Figure 3e) revealed that the corresponding *P*_S_‐*T* curves of BTZ showed a sharp decrease in *P*_S_ between 30°C and 50°C (Figure 3f), yielding a large ∂*P*/∂*T*. To quantitatively validate the pyroelectric enhancement, the coefficient was also calculated from the derivative of *P*_S_‐*T* curves. The calculated value for BTO was ∼486 µC m^−2^ K^−1^, which matched the direct measurement (∼450 µC m^−2^ K^−1^). For BTZ, the calculated value reached ∼1522 µC m^−2^ K^−1^. Although this value was lower than the directly measured peak (3505 µC m^−2^ K^−1^) due to the limited temperature resolution of the discrete *P‐E* loop measurements, which underestimated the sharp derivative at the phase transition, it consistently confirmed the significant enhancement over BTO. Besides, to further bridge the gap between experimental observations and theoretical predictions, we employed phase‐field simulations to model the domain structures and *P‐E* loops of BTZ and BTO in nanoscale. The simulated *P‐E* hysteresis loops (Figure S5) showed good agreement with experimental results obtained from the bulk ceramics. The dielectric spectrum revealed the temperature dependence of the dielectric constant with a peak at *T*_C_. As shown in Figure 3g, Zr doping lowered the *T*_C_ to 64°C, enhancing pyroelectric performance at a lower temperature. In comparison to other systems, such as bismuth layer‐structured ferroelectrics (BLSF), tungsten bronze, and other perovskites [28, 29, 30, 31, 32, 33, 34], the BTZ system in this study demonstrated a superior pyroelectric coefficient (Figure 3h).

The experimental findings were further interpreted using thermodynamic analysis, considering multiphase coexistence. Both Landau free‐energy modeling and phase‐field simulations were applied to probe phase stability at ambient and elevated temperatures (40°C). At room temperature, a narrow energy gap of ∼6 J cm^−3^ was found among R, O, and T phases in BTZ (Figures S6). Such a small gap indicated reduced polarization anisotropy and lowered transition barriers (Figure 3i). As the temperature increased, the energy gap further decreased to ∼4 J cm^−3^, indicating heightened sensitivity of the domain structure to thermal fluctuations (Figure S6c). Moreover, phase‐field simulations revealed striking differences between BTO and BTZ. While BTO featured large ferroelectric domains, BTZ exhibited nanoscale coexistence of R, O, and T phases distributed randomly as 8R, 6T, and 12O states with polarization vectors interpenetrating (Figure 3j). This nanodomain configuration allowed for multiple polarization‐rotation pathways, suggesting that thermal fluctuations can readily induce polarization switching owing to the reduced energy landscape.

### 2.3 Photo‐Pyroelectric Effect

**FIGURE 4:**
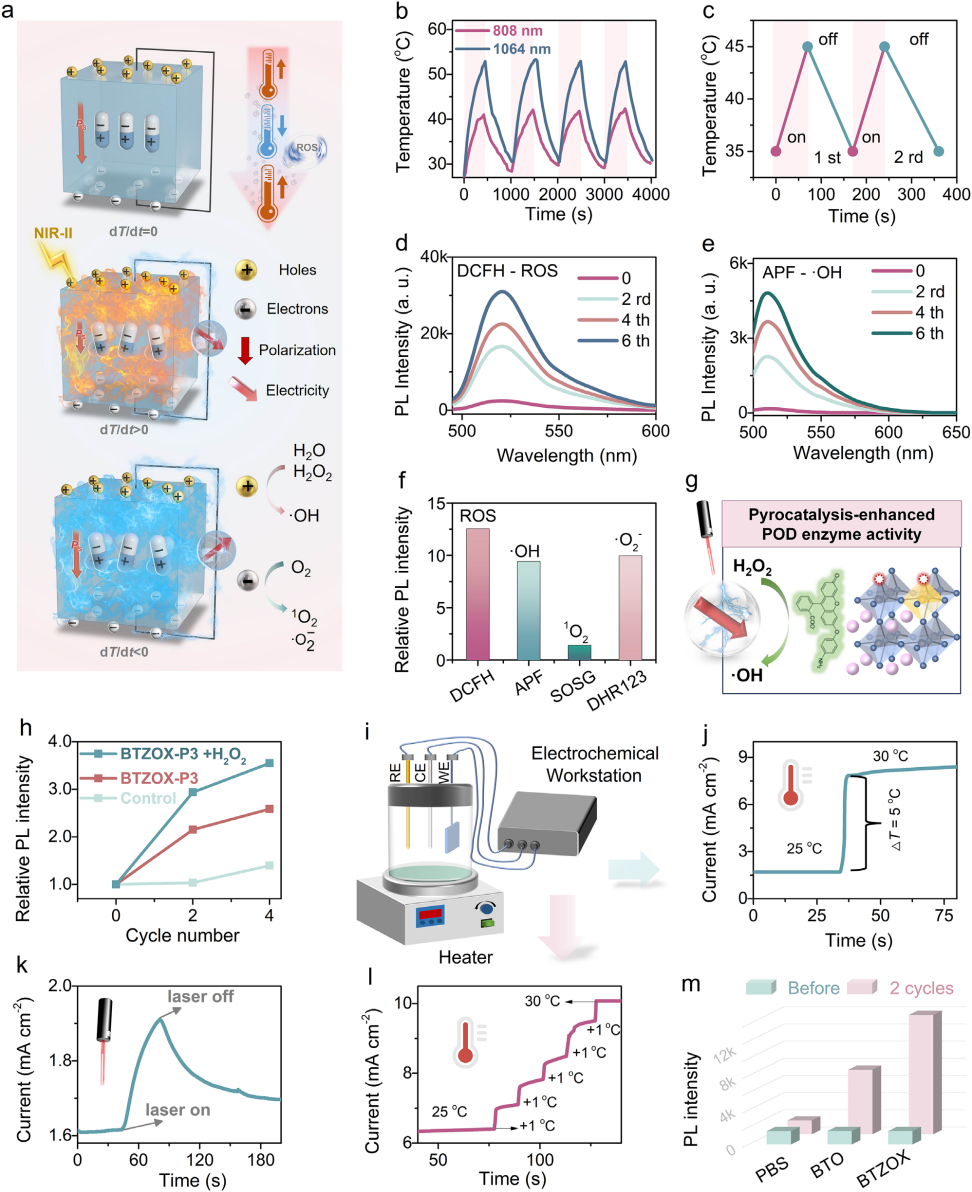
Photo‐pyroelectric effect of BTZOX‐P3 NCs. (a) Schematic of the light‐to‐thermal‐to‐electricity energy conversion and NIR‐activated pyrocatalysis. (b) Photothermal heating curves of BTZOX‐P3 dispersion (200 µg mL^−1^ in 0.01 m PBS) by 808 nm and 1064 nm laser irradiation (1.0 W cm^−2^). (c) Photothermal heating/cooling cycles of BTZOX‐P3 over two cycles. The generation of (d) ROS and (e) •OH of BTZOX‐P3 after several NIR‐induced heating/cooling cycles was detected by DCFH and APF probe, respectively. (f) Relative PL intensity after the 6th heating/cooling cycles using different fluorescence probes. (g) Schematic of pyrocatalysis‐enhanced POD‐like enzyme activity of BTZOX‐P3. (h) Relative PL intensity of BTZOX with or without H_2_O_2_ using APF probe indicating the generation of •OH. (i) Schematic of the electrochemical workstation setup. (j) Pyroelectric current of BTZOX‐P3 by a sudden rise of 5°C in temperature. (k) Pyroelectric current of BTZOX‐P3 under 1064 nm laser on/off irradiation. (l) Pyroelectric current of BTZOX‐P3 by a rise in temperature step‐by‐step. (m) PL intensity of DCFH incubated with BTO or BTZ dispersion after two heating/cooling processes (35°C–45°C).

Building on the preceding analysis, the improvement of the pyroelectric coefficient was expected to reinforce catalytic efficiency. Consequently, the pyroelectric catalytic properties of BTZOX‐P3 NCs were systematically examined. As depicted in Figure 4a, when exposed to a 1064 nm laser, BTZOX‐P3 underwent a heating–cooling cycle, during which non‐equilibrated polarization charges accumulated, giving rise to an intrinsic electric field. This field served as the driving force for surface electrochemical reactions, thereby facilitating the formation of ROS. The photothermal response of BTZOX‐P3 dispersions was investigated under irradiation at both 808 and 1064 nm (1.0 W cm^−2^). As shown in Figure 4b, the temperature increase was substantially higher under 1064 nm excitation, with a rise of ∼30°C within 6 min. In contrast, PBS, BTO, and BTZ dispersions displayed negligible changes, confirming that oxygen vacancies in BTZOX‐P3 efficiently convert NIR energy into heat. The heating behavior was further found to correlate with particle concentration (Figure S7). After 70 s of irradiation, the temperature of the BTZOX‐P3 solution increased by ∼10°C, followed by cooling back to 35°C within 100 s, completing one thermal cycle (Figure 4c). ROS production during repeated thermal cycles was tracked with the fluorescent probe 2′,7′‐dichlorofluorescein (DCFH). The intensity of fluorescence rose markedly after two cycles (Figure 4d), confirming cumulative ROS formation during heating/cooling. Additional probes, such as aminophenyl fluorescein (APF) for •OH, singlet oxygen sensor green (SOSG) for ^1^O_2_, and dihydrorhodamine 123 (DHR123) for •O_2_^−^, were employed (Figure 4e,f; Figures S8–S10). Results indicated that •OH and •O_2_^−^ were the predominant radicals generated, underscoring their central role in the catalytic process. Moreover, BTZOX‐P3 displayed peroxidase (POD)‐mimicking activity (Figure 4g). In the presence of H_2_O_2_, •OH generation was amplified by 1.37‐fold (Figure 4h), suggesting that NIR‐II‐stimulated pyroelectric activity not only triggered pyroelectric catalysis but also accelerated Fenton‐like pathways, leading to a rapid burst of ROS capable of promoting tumor cell apoptosis.

Electrochemical analysis performed with a workstation further elucidated this process (Figure 4i). Pyro‐current signals were recorded during heating–cooling cycles without light irradiation, revealing sharp increases in current with small temperature changes of 1°C–5°C (Figure 4j,l). Under 1064 nm irradiation, a pronounced pyroelectric current was observed (Figure 4k), arising from sequential conversion of light to heat and then to electricity. ROS formation through pure PCE was also confirmed by DCFH fluorescence in the absence of NIR irradiation (Figure 4m; Figure S11a). Comparisons with pristine BTO demonstrated that BTZ produced approximately twice as much ROS within the physiologically relevant 35°C–45°C range, attributable to its superior PCE (Figure S11b,c).

### 2.4 Mechanism for Enhanced Pyrocatalysis Performance

**FIGURE 5:**
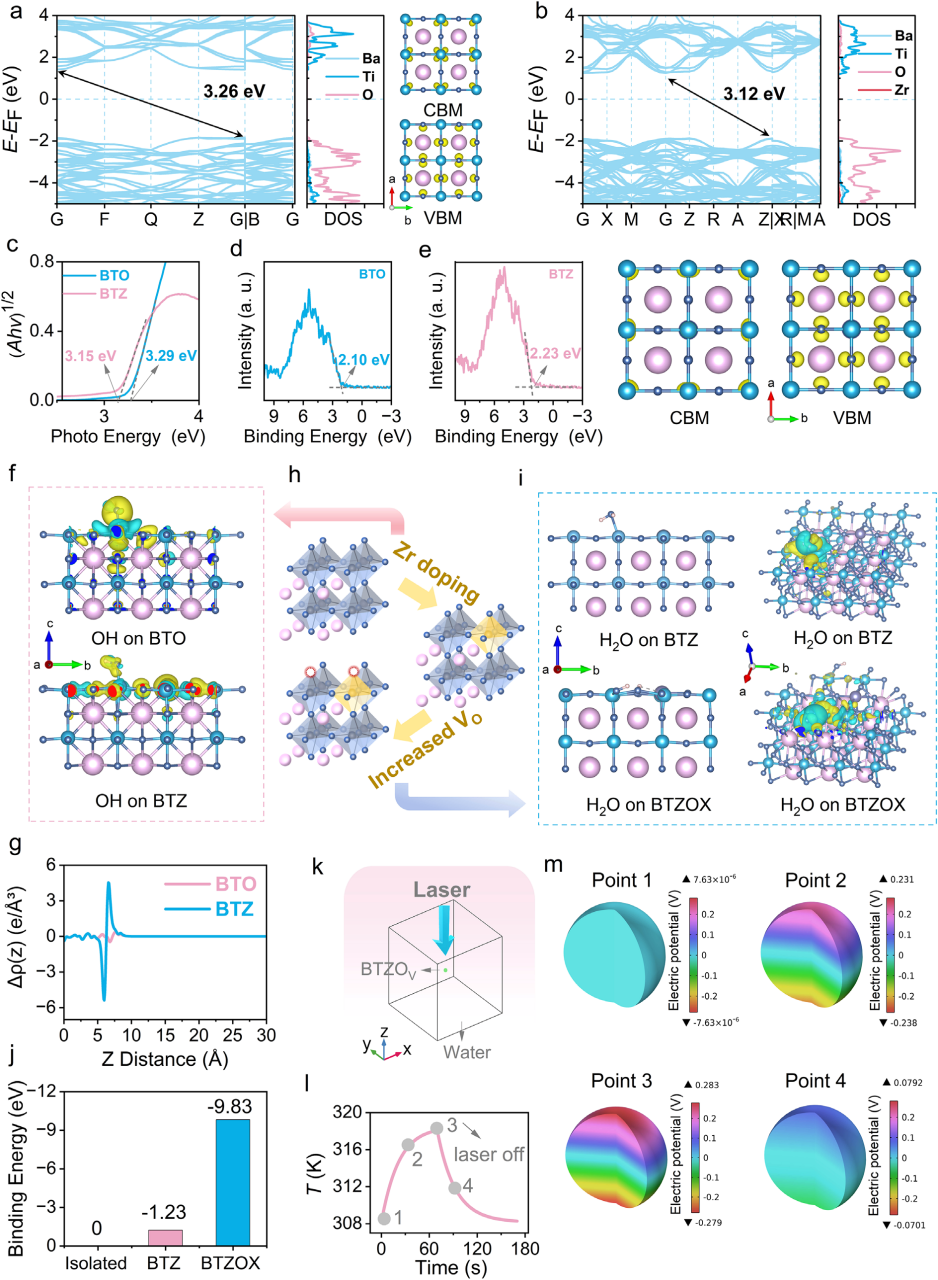
Electronic and interfacial structure of BTZ by DFT calculation. Orbital projected electronic band structure and projected density of states for (a) BTO and (b) BTZ. VBM and CBM orbital arrangements were illustrated. (c) Tauc plots of BTO and BTZ. VB‐XPS spectra of (d) BTO and (e) BTZ. (f) Charge density difference of OH adsorbed on BTO and BTZ. (g) The planar‐averaged charge density difference of BTO‐OH and BTZ‐OH. (h) Structure of BTZOX via Zr doping and oxygen vacancy design. (i) Charge density difference of H_2_O adsorbed on BTZ and BTZOX. (j) Adsorption energy of H_2_O on BTZ and BTZOX. (k) Schematic of the FEM simulation. (l) Selected temperature stage in the periodic thermal cycles, and (m) the corresponding distribution of pyroelectric potential of BTZOX‐P3 NPs by finite element modelling.

To gain insight into the enhanced pyrocatalytic effect of BTZOX‐P3, theoretical calculations were performed. First, the energy band structure of BTO and BTZ was analyzed using DFT calculations (Figure 5a,b). Zr incorporation caused a notable redistribution of charge density at the conduction band minimum (CBM), which reduced electron binding energy and facilitated electron migration and transfer during catalysis. This increased electron mobility allowed more efficient participation in catalytic cycles, enhancing overall catalytic conversion. Moreover, Zr doping lowered the CBM energy level, narrowing the bandgap from 3.26 to 3.12 eV. The reduced bandgap enabled electron‐hole pair generation at lower energies, improving charge carrier generation efficiency under thermal fluctuations in pyrocatalysis. Experimental confirmation of the bandgap narrowing was obtained via UV‐Vis diffuse reflectance spectroscopy (Figure S12), and Tauc plots indicated an optical bandgap of 3.29 eV for BTO and 3.15 eV for BTZ (Figure 5c), a reduction of 0.14 eV, which closely matched DFT predictions. The valence band (VB) of BTO and BTZ was determined by VB‐XPS (Figure 5d,e). Using the equation [35]:

(2) $$E_{\mathrm{NHE}}/V=\Phi +E_{f}-4.44$$

where *E*_NHE_ was the standard electrode potential, and Φ was the work function of XPS (4.2 eV in this work), the VBM of BTO and BTZ was calculated to be 1.86 and 1.99 V (*vs*. NHE), respectively. Consequently, the CBM of BTO and BTZ were −1.43 and −1.16 V, respectively. This energy band structure of BTZ was favorable for efficient •OH and •O_2_^−^ generation, as the VBM exceeded the redox potential of OH^−^/•OH (1.5 V), while the CBM was lower than the O_2_/•O_2_^−^ redox potential (−0.33 V).

In catalytic reactions, the adsorption behavior between surface active sites and reactant substrates was crucial. To investigate this interaction at the electronic level, we computed the charge density difference for hydroxyl (OH^−^) adsorption on BTO and BTZ surfaces (Figure 5f). The planar‐averaged charge density difference analysis (Figure 5g) revealed that the Zr‐OH system exhibited larger oscillation amplitudes perpendicular to the surface, reflecting a charge redistribution induced by Zr doping from in‐plane (parallel) charge accumulation to steeper out‐of‐plane gradients. This anisotropic charge rearrangement optimized the adsorption energy, enhancing the interfacial charge transfer necessary for catalytic activity. Beyond cationic doping, we further engineered interfacial oxygen vacancies as catalytically active sites (Figure 5h) and studied the adsorption behavior of H_2_O on BTZ and BTZOX. DFT calculations revealed two distinct H_2_O adsorption modes: molecular adsorption on BTZ surface (BTZ+H_2_O→BTZ‐H_2_O, adsorption energy: −1.23 eV, Figure 5i); dissociative adsorption at an oxygen vacancy site (BTZ‐V_O_+H_2_O→BTZ‐OH+BTZ‐H, adsorption energy: −9.83 eV, Figure 5j). During dissociative adsorption, the water molecule cleaves at the vacancy, with the OH^−^ filling the vacancy and H^+^ binding to a neighboring surface oxygen. The oxygen vacancy promoted dissociative adsorption, strengthening the binding energy by 8.60 eV. In addition, the planar‐averaged charge density difference analysis also revealed that the V_O_‐H_2_O system exhibited larger oscillation amplitudes perpendicular to the surface (Figure S13). This confirmed that surface oxygen vacancies served as crucial active sites for water molecule activation, further enhancing the generation of •OH under pyroelectric carriers.

Furthermore, the pyroelectric characteristics of BTZOX‐P3 NCs were further examined using finite element method (FEM) simulations. As illustrated in Figure 5k, a computational model was constructed, consisting of a cubic water medium (3000 nm in size) with a spherical BTZOX‐P3 particle positioned at its center. A 1064 nm laser beam (1.0 W cm^−2^) was directed along the z‐axis to simulate irradiation conditions. Heat transfer from the BTZOX‐P3 particle into the surrounding aqueous environment was incorporated to evaluate the evolution of temperature (Figure 5l). During both heating and cooling stages, the distribution of pyroelectric potential was visualized (Figure 5m). The potential difference across the polar facets of BTZOX‐P3 closely followed the variation in temperature. At the highest temperature point, the open‐circuit voltage reached a maximum of 0.562 V. These findings indicate that the fluctuation of electric potential with thermal change in BTZOX‐P3 provides sufficient driving force to sustain continuous redox reactions at the material's surface.

**FIGURE 6:**
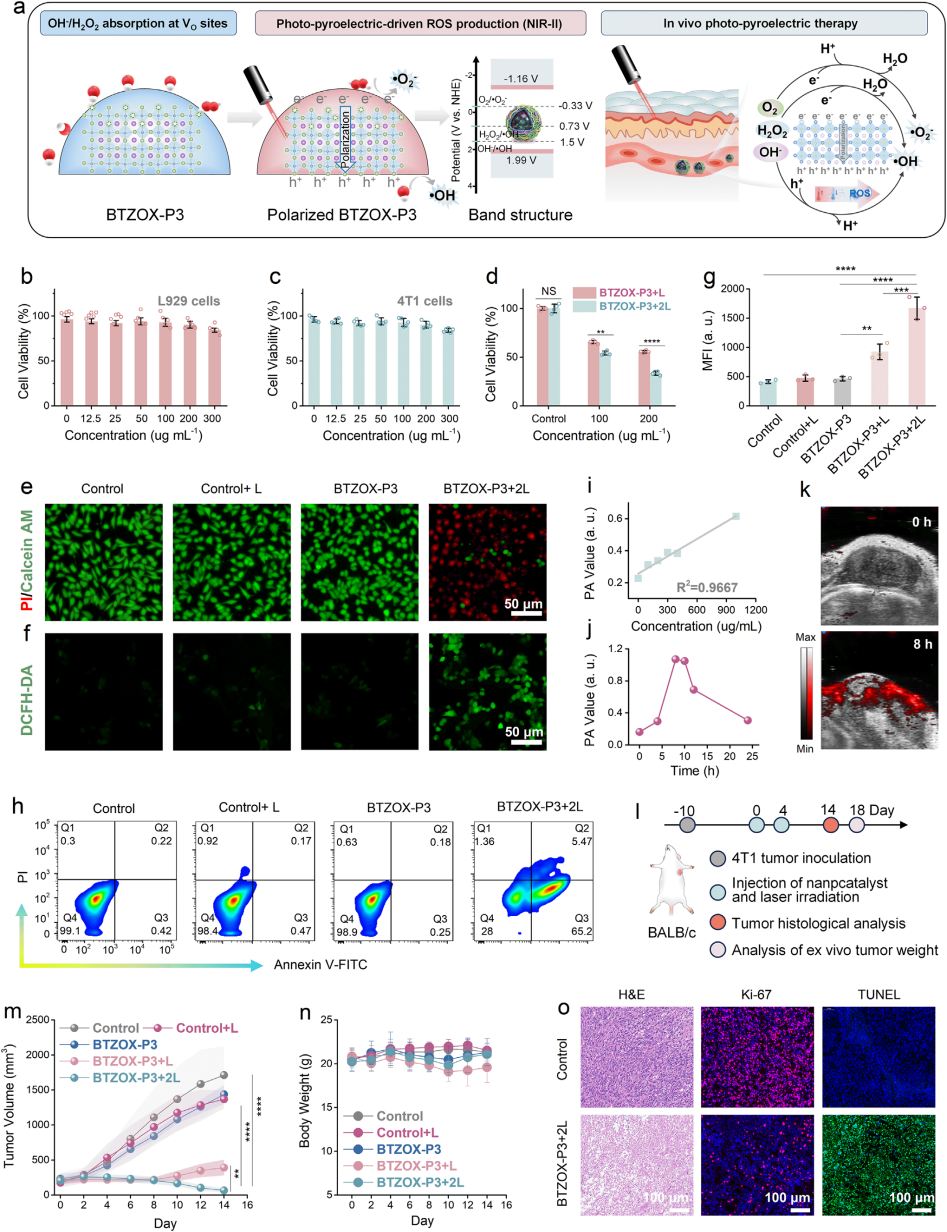
In vitro and in vivo photo‐pyroelectric therapy. (a) Schematic illustration of the pyroelectric‐catalytic ROS generation mechanism of BTZOX‐P3 and in vivo photo‐pyroelectric therapy. Cell viability of (b) L929 and (c) 4T1 cancer cells following incubation with BTZOX‐P3 NCs for 24 h (*n* = 5 biologically independent samples). (d) Cell viability of 4T1 cancer cells after being treated with BTZOX‐P3 NCs under 1064 nm laser exposure (*n* = 4 biologically independent samples). *P*‐values: ^**^ < 0.01 and ^****^ < 0.0001. (e) CLSM images of 4T1 cancer cells stained with calcein AM (green)/PI (red) after different treatments. (f) DCFH‐DA fluorescence images of 4T1 cancer cells with diverse treatments. (g) Flow cytometry analysis of intracellular ROS stained by DCFH‐DA (*n* = 3 biologically independent samples). *P*‐values: ^**^ < 0.01, ^***^ < 0.001, and ^****^ < 0.0001. (h) Flow cytometry and Annexin V‐FITC/PI‐stained 4T1 cancer cells after different treatments. i In vitro PA signal intensity of different BTZOX‐P3 concentrations. (j) Time‐dependent PA images within tumor sites post‐injection of BTZOX‐P3 and (k) PA signal intensity in tumor regions at different times. (l) Schematic of the therapeutic process. (m) Tumor volume of diverse treatment groups. *n* = 6 biologically independent samples, *P*‐values: ^**^< 0.01, ^****^< 0.0001. (n) Body weight growth curves of all groups after various treatments, *n* = 6 biologically independent samples. (o) H&E, Ki‐67, and TUNEL staining of tumor tissues in diverse treatment groups.

To clarify the underlying principle of the enhanced photo‐pyroelectric catalytic activity, the ROS generation mechanism was schematically illustrated in Figure 6a. In the physiological microenvironment, the abundant oxygen vacancies on the BTZOX‐P3 surface served as specific active sites, effectively adsorbing reactants such as H_2_O_2_, H_2_O, and OH^−^. Under NIR‐II laser cycling, the periodic temperature fluctuation triggered the pyroelectric effect in the BTZOX‐P3, establishing a built‐in electric field that drove the separation and migration of electrons (e^−^) and holes (h^+^) to the surface. The CBM was located at −1.16 V (vs. NHE), which was significantly more negative than the potentials required for O_2_ reduction to •O_2_^−^ (−0.33 V, vs. NHE) and the one‐electron reduction of H_2_O_2_ to •OH (+0.73 V, vs. NHE) [36]. Thus, the photogenerated electrons can spontaneously trigger these reduction reactions. Besides, the VBM was located at +1.99 V. Consequently, the photogenerated holes can capture the adsorbed OH^−^ (approx. +1.5 V for surface oxidation) to generate •OH. Collectively, the combined effects of band‐structure modulation and interface optimization were found to significantly amplify the pyrocatalytic response under periodic temperature variation, ultimately yielding an orders‐of‐magnitude increase in ROS generation.

### 2.5 In Vitro Anticancer Performance

Motivated by the outstanding pyrocatalytic behavior and the elevated generation of ROS exhibited by BTZOX‐P3 NCs, their therapeutic potential was further evaluated in vitro. Cytocompatibility was first examined through the Cell Counting Kit‐8 (CCK‐8) assay using murine fibroblast cells (L929, Figure 6b) and 4T1 breast carcinoma cells (Figure 6c). Exposure to BTZOX‐P3 at a concentration of 300 µg mL^−1^ resulted in negligible cytotoxic effects, verifying excellent cellular compatibility. Under irradiation with a 1064 nm laser for 5 min (abbreviated as 1L), the medium temperature rose from 35°C to 45°C, which led to a roughly 50% reduction in 4T1 cell survival (Figure 6d). Repetition of the irradiation for two cycles (2L) further intensified the cytotoxic response, highlighting the pronounced photo‐pyroelectric effect. Live/dead staining supported these findings: pronounced cell mortality was observed after 1L treatment (red fluorescence), and almost total ablation occurred following 2L exposure (Figure 6e; Figure S14a). To probe intracellular ROS production, cells were treated with 2′,7′‐dichlorodihydrofluorescein diacetate (DCFH‐DA). Upon oxidation by ROS, the probe emitted green fluorescence (Figure 6f; Figure S14b). Weak signals were detected when cells were exposed to BTZOX‐P3 alone or laser irradiation alone, whereas the combined treatment produced strong green fluorescence, which became more intense after two cycles, indicating substantial ROS induction. Flow cytometric analysis of DCFH‐DA fluorescence corroborated this enhancement in the BTZOX‐P3+2L group (Figure 6g; Figure S15). Apoptotic pathways were further assessed using Annexin V and propidium iodide (PI) dual staining (Figure 6h; Figure S14c). The four quadrants of Annexin V/PI plots correspond to necrosis (Q1), early apoptosis (Q2), late apoptosis (Q3), and viable cells (Q4). Compared with control, BTZOX‐P3+L and BTZOX‐P3+2L groups showed a substantial increase in apoptotic populations, with early apoptosis rising to 42.57% and late apoptosis reaching 70.67%, consistent with the CCK‐8 results. Finally, hemolysis experiments demonstrated that BTZOX‐P3 did not induce significant red blood cell damage, thereby excluding major concerns regarding blood compatibility (Figure S16).

### 2.6 In Vivo Therapeutic Performance

Owing to the strong NIR absorption and efficient photothermal conversion, BTZOX‐P3 served as a robust contrast agent for photoacoustic (PA) imaging. A clear PA response was detected, which displayed a concentration‐dependent linear increase (Figure 6i; Figure S17a). After systemic administration through intravenous injection, PA signals were monitored within tumor tissue and reached their maximum intensity approximately 8 h post‐injection (Figure 6j,k; Figure S17b). The accumulation of BTZOX‐P3 at the tumor site can be attributed to the enhanced permeability and retention (EPR) effect [37]. These observations suggested that BTZOX‐P3 had considerable potential for PA‐guided therapeutic interventions. In subsequent animal experiments, 4T1 tumor‐bearing mice were randomly divided into five treatment cohorts, and the study design followed the scheme illustrated in Figure 6l. Animal studies were approved by the Animal Ethics Committee of Fujian Normal University (20230032). Based on the timing established by PA imaging, laser irradiation at 1064 nm was applied to tumor regions 8 h after injection. Thermal imaging revealed that tumor surface temperature remained under 45°C throughout the procedure (Figure S18). Tumor progression and body weight were recorded every two days. Notably, groups treated with BTZOX‐P3+L or BTZOX‐P3+2L exhibited pronounced tumor suppression (Figure 6m), with the latter achieving a growth inhibition rate of 97.7% (Figure S19). The survival curves of the mice were recorded (Figure S20), showing that the groups treated with BTZOX‐P3+L or BTZOX‐P3+2L extended the period of survival. Resected tumors were subsequently photographed and weighed for further evaluation (Figure S19). No significant loss of body weight was observed in any group, indicating acceptable systemic safety (Figure 6n).

To further evaluate biosafety, blood biochemical markers including creatine kinase (CK), lactate dehydrogenase (LDH), uric acid (UA), creatinine (CR), aspartate aminotransferase (AST), and alanine aminotransferase (ALT) were measured on day 7th. All values remained comparable to the control group (Figure S21). Histological examinations of major organs (heart, liver, spleen, lungs, kidneys) using hematoxylin and eosin (H&E) staining revealed no pathological abnormalities, supporting the absence of systemic toxicity (Figure S22). Histological analyses of tumor tissues provided further mechanistic insights. H&E staining of samples from BTZOX‐P3+L and BTZOX‐P3+2L groups displayed extensive necrotic damage, characterized by cytoplasmic vacuolation, intercellular gaps, karyopyknosis, and plasmorrhexis, with the latter group showing more severe injury (Figure S23). Additionally, Ki‐67 immunostaining and TUNEL assays (Figure 6o; Figure S24) confirmed that the combined treatment markedly reduced proliferative activity while significantly promoting apoptosis in tumor cells. Taken together, these comprehensive results demonstrated that BTZOX‐P3 nanocatalysts possessed strong antitumor efficacy along with excellent biocompatibility, highlighting their promise as photo‐pyroelectric therapeutic agents activated in the NIR‐II window. This co‐engineering strategy combining polarization tuning and interface activation offers a generalizable blueprint for next‐generation pyroelectric nanotherapeutics.

## 3 Discussion

The good therapeutic efficacy of BTZOX‐P3 stemmed from the precise co‐engineering of intrinsic lattice dynamics and interfacial defect chemistry. Our results demonstrated that the 15% Zr‐doping is not merely a structural modification but a critical tuner of the thermodynamic energy landscape. By shifting the Curie temperature to the physiological window and inducing a multiphase coexistence, we maximized the polarization sensitivity to temperature fluctuations (*dP*/*dT)*, resulting in a large pyroelectric coefficient of 3505 µC m^−2^ K^−1^. This intrinsic enhancement ensured a robust driving force for charge carrier separation under mild thermal stimuli. Complementarily, the introduction of oxygen vacancies played a dual role: it narrowed the bandgap to extend optical absorption into the deep‐penetrating NIR‐II region and created unsaturated surface sites that significantly lower the adsorption energy for H_2_O and OH─. This synergy overcame the two main bottlenecks of conventional pyrocatalysis: low charge generation efficiency and sluggish surface reaction kinetics.

The ROS generation mechanism of BTZOX‐P3 represented a cascade of energy conversion events. Upon 1064 nm laser irradiation, the non‐radiative relaxation of excited electrons in the defect‐rich lattice generated local heat, creating rapid temporal temperature gradients. Unlike conventional photocatalysis, which relies solely on photon energy to excite electron‐hole pairs, our system primarily exploits this thermal fluctuation. The temperature change altered the spontaneous polarization intensity of the BTZOX‐P3 nanodomains, disrupting the surface charge balance and establishing a built‐in electric field. Thermodynamic band alignment analysis confirmed that the conduction band potential is sufficient to drive the reduction of O_2_ and H_2_O_2_, while the defect‐modulated valence band facilitated the oxidation of OH^−^. The pyroelectric field effectively suppressed the recombination of these thermally‐induced carriers, ensuring a continuous and high‐flux output of reactive oxygen species (•OH and •O_2_^−^) for tumor ablation.

Recently, thermal‐mediated catalytic therapy has emerged as a cutting‐edge frontier in oncology, where both thermoelectric and pyroelectric effects show immense potential in harvesting thermal energy for ROS generation. A number of studies have highlighted the efficacy of thermoelectric strategies, which utilize the Seebeck effect driven by spatial temperature gradients [38, 39, 40, 41, 42]. For instance, Yuan et al. reported magnetically triggered thermoelectric heterojunctions for synergistic therapy [38], and they also developed self‐triggered nanoheterojunctions that utilize chemical heat from CaO_2_ to drive catalysis [42]. Similarly, advanced heterojunction designs have been employed to optimize carrier transport for surgical adjuvant treatment [41, 43]. While both strategies rely on thermal fields, their driving mechanisms are distinct. Thermoelectric catalysis depends on a spatial temperature difference across the material to drive carrier diffusion (the Seebeck effect). In contrast, the pyroelectric catalysis presented in this work exploits temporal temperature variations (*dT/dt*) to induce polarization changes (*dP/dT*). This unique feature allows pyroelectrics to respond rapidly to high‐frequency laser pulses without necessarily requiring a large macroscopic temperature gradient across the nanoparticle. Despite these mechanistic differences, the future evolution of both thermoelectric and pyroelectric nanotherapeutics converges on three key directions: (i) smart triggering: shifting from passive heating to active, multi‐stimuli responsive systems (e.g., magnetic, chemical, or enzymatic triggers); (ii) heterojunction engineering: constructing built‐in fields (p‐n junctions, Schottky barriers) to prolong carrier lifetime; and (iii) synergistic modes: integrating thermal catalysis with immunotherapy or starvation therapy.

The clinical significance of this study lies in its one‐stone‐two‐birds strategy. Traditional photothermal therapy often requires high temperatures (>50°C) to kill tumors, posing risks to adjacent healthy tissues. In contrast, our photo‐pyroelectric strategy operates efficiently under mild hyperthermia (<45°C). The thermal energy is not just for cooking the tumor, but is primarily converted into chemical weapons (ROS). This implies that deep‐tissue tumor eradication can be achieved with minimal thermal damage, offering a safer modality for heat‐sensitive regions. Despite the promising results, we acknowledge certain limitations. First, the current study utilized a rapid‐growth 4T1 murine model, which precluded long‐term biological safety monitoring (e.g., over several months). Future studies should employ orthotopic or slower‐growing models to strictly validate long‐term biocompatibility. Second, while the EPR effect enables passive targeting, the accumulation efficiency can be further improved. Future work will explore surface functionalization with targeting ligands or adaptive coatings to further enhance the specificity and clinical translation potential of this pyroelectric platform.

## 4 Conclusion

In this study, we present a robust strategy for engineering high‐performance pyroelectric nanocatalysts by simultaneously modulating intrinsic ferroelectric polarization and interfacial reactivity. Through Zr doping, the *T*_C_ of BTZ was precisely shifted toward the physiological window, accompanied by phase convergence and enhanced polarization sensitivity to temperature fluctuations. This led to a high pyroelectric coefficient *ρ* of 3505 µC m^−2^ K^−1^, over 8 times higher than pristine BTO, enabling highly efficient thermal‐to‐electrical energy conversion under mild thermal cycling. To address the second bottleneck of surface catalytic inefficiency, we introduced oxygen vacancies, enhancing NIR‐II photothermal absorption and creating abundant unsaturated active sites for dissociative H_2_O adsorption. DFT calculations corroborated that the synergistic interplay between bandgap narrowing, anisotropic charge redistribution, and defect‐induced water activation pathways significantly boosted ROS generation efficiency. Upon NIR‐II irradiation, BTZOX‐P3 achieved spatiotemporally controlled, repeatable photo‐pyroelectric ROS bursts, triggering effective tumor cell apoptosis both in vitro and in vivo, while maintaining minimal systemic toxicity. The dual benefits of precise thermal responsiveness and amplified redox reactivity position BTZOX‐P3 as a promising platform for ROS‐mediated oncotherapy. Our findings not only advance the frontier of dynamic catalysis in biomedical settings but also pave the way for precision ROS engineering in broader therapeutic contexts.

## 5 Experimental Section

### 5.1 Chemicals

Tetrabutyl titanate, zirconyl chloride octahydrate, ammonium hydroxide, barium hydroxide monohydrate, dimethylaminopyridine, 2‐bromo‐isobutyryl bromide, tetrahydrofuran, trifluoroacetic acid, polyethyleneimine, and polyethyleneimine were purchased from Shanghai Aladdin Bio‐Chem Technology Co., Ltd. Ethanol and acetic acid were from Sinopharm Chemical Reagent Co., Ltd. Phosphate buffered solution (PBS, pH 7.4), DCFH‐DA, SOSG, DHR123, APF, calcein acetoxymethyl ester (Calcein‐AM), and propidium iodide (PI) were purchased from Sigma–Aldrich (Shanghai) Trading Co., Ltd. CCK‐8 was purchased from Dojindo Laboratories. Dulbecco's Modified Eagle's Medium (DMEM) medium, fetal bovine serum (FBS), penicillin, and streptomycin were purchased from Gibco‐Invitrogen Corp. Annexin V‐FITC was purchased from Beyotime Biotechnology Co., Ltd. Polyethylene glycol monomethyl ether, triethylamine, dichloromethane, anhydrous sodium sulfate, cuprous bromide, tert‐butyl acrylate, and ether were from Shanghai Macklin Biochemical Technology Co., Ltd. Pentamethyldiethylenetriamine, N, N‐dimethylformamide, 1‐Ethyl‐3‐(3‐dimethylaminopropyl)carbodiimide, and N‐Hydroxysuccinimide were from Shanghai energy‐chemical Technology Co., Ltd.

### 5.2 Synthesis of BTZOX NCs

The hydrothermal method was employed to synthesize Ba(Ti_0.85_Zr_0.15_)O_3_ nanoparticles. 2.92 mL of Ti(C_4_H_9_O)_4_ was added to 10 mL of ethanol and stirred for 30 min. Following this, 0.483 g of ZrOCl_2_·8H_2_O was introduced, and the mixture was stirred for 30 mins. NH_3_·H_2_O was incrementally added until no more deposits formed. The resulting suspension was then transferred into a Teflon‐lined autoclave. Simultaneously, 4.3692 g of Ba(OH)_2_·H_2_O was dissolved in 20 mL ultra‐pure (UP) water. After complete dissolution, this solution was combined with the suspension. Subsequently, the Teflon‐lined autoclave underwent heating at 180°C for 48 h. The obtained products underwent successive washing in acetic acid, ethanol, and deionized water, followed by drying at 80°C for 24 h. For further processing, 150 mg of the resulting Ba(Ti_0.85_Zr_0.15_)O_3_ powder was mixed with 300 mg of sodium borohydride and ground for 20 mins. This mixture underwent calcination in a tube‐heating furnace at 425°C for 90 mins under an argon atmosphere. After centrifugation and washing to remove excess sodium borohydride, the sample was dried to yield BTZOX.

### 5.3 Synthesis of PAA‐PEG

(a) The synthesis of PEG‐Br. In the atmosphere of nitrogen, polyethylene glycol monomethyl ether (1 g, 0.2 mmol), triethylamine (44.52 mg, 0.44 mmol), dimethylaminopyridine (2.44 mg, 0.02 mmol), and 2‐bromo‐isobutyryl bromide (91.96 mg, 0.4 mmol) were dissolved in 15 mL of dichloromethane solution. After stirring overnight, it was washed with water 2–3 times and dried with anhydrous sodium sulfate. Finally, the product was obtained after the ether deposition 3 times. (b) The synthesis of PEG‐TBA. In the atmosphere of nitrogen, PEG‐Br (500 mg, 0.1 mmol), cuprous bromide (43.04 mg, 0.3 mmol), tert‐butyl acrylate (1.28 g, 10 mmol), and pentamethyldiethylenetriamine (PMEDTA) (51.99 mg, 0.3 mmol) were added into 1.4 mL anhydrous N, N‐dimethylformamide solution, stirred overnight at 60°C, and stopped polymerization in contact with atmosphere under ice bath. After being diluted with tetrahydrofuran solution, the alumina column was perwatered, the filtrate was spun dry, and the product was settled with ether three times. (c) The synthesis of PEG‐PAA. In the atmosphere of nitrogen, PEG‐TBA was dissolved in 1 mL of anhydrous dichloromethane solution, and 1 mL of trifluoroacetic acid solution was added drop by drop and stirred at room temperature overnight. At the end of the reaction, the product was obtained by settling three times with ether and drying.

### 5.4 Synthesis of BTZOX‐P3 NCs

By dispersing 30 mg BTZOX in UP water and ultrasound for 40 min, solution A was obtained. 40 mg PEI was dissolved in pure water and added to solution A drop by drop. The BTZOX‐PEI was obtained by centrifugation after ultrasound for 30 min. 30 mg PEG‐PAA was dissolved in UP water, and 30 mg BTZOX‐PEI, 7.5 mg EDC, and 7.5 mg NHS were added to the PEG‐PAA solution and stirred for 2 h. After centrifuging and washing with PBS 3 times, BTZOX‐PEI‐PAA‐PEG (BTZOX‐P3) was obtained. The product was dispersed in 1× PBS solution for preservation.

### 5.5 Characterization

XRD patterns were obtained employing a DY1602 X‐ray diffractometer (Empyrean, Malvern Panalytical). The Rietveld refinement of BTZ powder XRD data was conducted employing MAUD software. High‐resolution images and corresponding lattice fringes were photographed utilizing HRTEM (Talos F200i, FEI). Raman spectroscopy with a 532 nm excitation source (Invia Reflex, Renishaw) was used to obtain Raman spectra. XPS (K‐Alpha+, Thermo Fisher) was used to obtain the chemical state, and the valence band was determined via. Surface oxygen vacancies were confirmed using electron paramagnetic resonance (EPR) spectroscopy (A300, Bruker). The ultraviolet‐visible‐near‐infrared (UV–vis–NIR) diffuse reflectance spectroscopy (Cary 7000, Agilent) was used to analyze optical absorption properties with BaSO_4_ as the reference. A nanoparticle size and zeta potential analyzer (Zetasizer Nano ZS) was used to obtain nanoparticle size and zeta potential. To investigate the ferroelectric domain switching behavior at the nanoscale, PFM measurements were conducted on the dispersed BTO and BTZOX nanoparticles using an atomic force microscope (MFP‐3D Origin+, Oxford Instruments, UK). To evaluate the macroscopic electrical properties, the synthesized BTO and BTZ nanoparticles were pressed into pellets and sintered at 1400°C for 3 h to obtain densified polycrystalline ceramics. Silver paste was applied to both sides of the ceramic pellets to serve as electrodes. The temperature‐dependent dielectric constant was measured using a precision LCR meter (TH2816A, Tonghui Electronic Co., Ltd., China). The polarization‐electric field (P‐E) hysteresis loops were recorded using a ferroelectric analyzer (TF Analyzer 2000, aixACCT Systems GmbH, Germany). The pyroelectric coefficient was determined using a dedicated pyroelectric coefficient test system (PCTS‐2000, Wuhan Yanhe Technology Co., Ltd., China) with a heating rate of 2°C min^−1^.

### 5.6 Calculations

The first principle calculations and molecular dynamics simulations are carried out based on density functional theory (DFT) using the Vienna Ab‐initio Simulation Package (VASP) [44] and PWMAT [45, 46] software. To accelerate HSE06 hybrid functional calculations, the band structure and density of states calculations were performed using PWMAT with SG15 pseudopotentials, a *k*‐point mesh of 4 × 4 × 4 for Brillouin zone sampling, and a plane wave cutoff energy of 40 Ryd. A 2 × 2 × 2 supercell was constructed to investigate the effects of doping on band structure and density of states.

A 3 × 3 × 2 supercell was constructed, and VASP software was used to calculate the adsorption behavior of OH and H_2_O. The exchange‐correlation functional was treated within the generalized gradient approximation (GGA) using the PBEsol functional [47], with the DFT‐D3 method included to account for van der Waals interactions. The projector augmented wave (PAW) method was employed to describe the electron‐ion interactions [48]. The plane wave cutoff energy was set to 520 eV. The non‐spherical contributions were included (LASPH = TRUE.) for an accurate description of the transition metal Ti electronic structure. The force convergence criterion was set to EDIFFG = −0.01 (residual force on each atom less than 0.01 eV/Å), and the electronic convergence criterion was set to EDIFF = 10^−5^ eV. For the molecular dynamics simulations, the on‐the‐fly machine learning force fields (MLFF) [49, 50] are first trained in a 3 × 3 × 3 perovskite supercell of BaTi_0.85_Zr_0.15_O_3_ structure containing 135 atoms to conduct the ab initio molecular dynamics (AIMD). In the training process, the simulations are executed in the NPT ensemble for 50 ps using the time step of 2 fs with an energy convergence criterion of 10^−4^ eV at 100, 200, 300, 400, and 500 K, respectively. Since the phase transition order is strongly dependent on the pressure in perovskite ferroelectrics and the current methods usually underestimate the phase transition temperature, a negative pressure of −2.8 GPa is used to ensure the phase transition temperature is generally consistent with experimental values. The root mean squared errors (RMSEs) of energies, forces, and stress of obtained MLFF predictions with respect to ab initio results for the training data are 3.12 × 10^−4^ eV per atom, 5.65 × 10^−2^ eV/Å, and 0.694 kB, respectively. After the training, the 6 × 6 × 6 perovskite supercell based on the generated MLFF is used to execute MD simulations with a total time of 50 ps and a time step 2 fs at constant temperatures of 280, 290, 300, 310, and 320 K, respectively.

### 5.7 Photothermal Properties

A 2 mL aqueous dispersion of BTZOX‐P3 (0–300 µg mL^−1^) was transferred into an EP tube. Subsequently, it underwent irradiation under an 808/1064 nm NIR laser at a power density of 1.0 W cm^−2^. Using an infrared camera (FLIR E8‐XT), the temperature of the dispersion was monitored. The control group, consisting of UP water, underwent the same treatment conditions.

### 5.8 Total ROS Detection by DCFH

In a typical procedure, 30 µL of DCFH (concentration: 30 µm) was combined with 3 mL of BTZOX‐P3 solution (200 µg mL^−1^). The resulting solution underwent exposure to 1064 nm irradiation (1.0 W cm^−2^) with a light cycle of 70 s on and 100 s off. Subsequently, 200 µL of the solution was extracted every 2 cycles and diluted to 3 mL with UP water. Following centrifugation, the fluorescence intensities of the supernatant at 520 nm were measured using a fluorescence spectrofluorometer (excitation wavelength: 489 nm).

### 5.9 Detection of ^1^O_2_/•OH/$\cdot O_{2}^{-}$ Generation

30 µL SOSG in DMSO (working concentration: 30 µm), 30 µL APF in DMF (concentration: 30 µm), or 30 µL DHR 123 in DMSO (concentration: 30 µm) was mixed with 3 mL BTZOX‐P3 solution (200 µg mL^−1^), respectively. The resulting solution underwent exposure to 1064 nm irradiation (1.0 W cm^−2^) with a light cycle of 70 s on and 100 s off. Subsequently, 200 µL of the solution was extracted every 2 cycles and diluted to 3 mL with UP water. Following centrifugation, the fluorescence intensities of the supernatant at 525 nm (SOSG), 530 nm (DHR123), and 515 nm (APF) were measured using a fluorescence spectrofluorometer.

### 5.10 Measurement of Pyroelectric Current

The pyroelectric current was determined using an electrochemical system (CHI‐660B, China). A Pt sheet was used as the counter electrode, and Ag/AgCl was the reference electrode. The working electrode, FTO glass, was prepared by spin‐coating BTZOX‐P3 NCs with Nafion and ethyl alcohol on the FTO glass and heating the electrode at 60°C for 1 h in a drying oven. The electrolyte used was a 0.5 m Na_2_SO_4_ solution. Pyroelectric responses to laser light (1064 nm, 1.0 W cm^−2^) were measured both with the light on and off in a black box. Additionally, the photoelectric response to thermal fluctuations was obtained by introducing a hot electrolyte into the system.

### 5.11 Finite Element Simulation

To simulate the pyroelectric behaviour of nanosphere material, a pyroelectricity multiphysics interface was employed in COMSOL 6.2. The pyroelectric potential *V* was evaluated by $V=p\cdot \Delta T\cdot \frac{a}{\varepsilon }$, where *ρ*, ∆*T*, a, and *ε* were the pyroelectric coefficient, the temperature difference, the characteristic size, and the dielectric constant of the sphere, respectively. The *ρ* and *ε* used in the simulation were 400 µC m^−2^ K^−1^ and 2400 according to the experiment result. The pyroelectricity interface fully coupled the electrostatics and the heat transfer, which was solved by the finite element method. Based on the concentration of the solution (300 µg mL^−1^ BTZOX‐P3 in water), a cubic model with a characteristic size of 3000 nm was used in the simulation. The sphere particle was located at the cubic center. To approach the actual situation in the experiment, the laser scattering was accounted for with a thermal flux of 1.0 W cm^−2^ in the heating stage (0–70 s), and the radiation from the surface to the ambient was also considered. As a result, variations in temperature and the pyroelectric potential were presented over time.

### 5.12 Landau Modeling

The domain structures were characterized using the spatial distribution of spontaneous polarization. The temporal evolution of the polarization was captured by the time‐dependent Ginzburg‐Landau (TDGL) equation $\frac{\partial P_{i}}{\partial t}+L\;\frac{\delta F}{\delta P_{j}}=0$. Here, *L* was a kinetic coefficient related to domain wall mobility, *F* was the total free energy of the system, and was the thermodynamic driving force for polarization evolution. The total free energy of a bulk system can be determined as follows:

(3) $$F=f_{bulk}\left(P\right)+f_{grad}\left(P\right)+f_{elastic}\left(P\right)+f_{elec}\left(P,E\right)$$

Herein, *F* included the bulk free energy *f_bulk_* (*P*), gradient energy *f_grad_* (*P*), elastic energy *f_elastic_* (*P*), and electrostatic energy *f_elec_* (*P*,*E*), where *E* was the applied static electric field. The bulk free‐energy density was expressed for zero strain as a six‐order polynomial expansion, which was *f_bulk_* = *α*_1_(*P*_1_2 + *P*_2_^2^ + *P*_3_^2^) + *α*_11_(*P*_1_^4^ + *P*_2_^4^ + *P*_3_^4^) + *α*_12_(*P*_1_^2^ *P*_2_^2^ + *P*_2_^2^ *P*_3_^2^ + *P*_1_^2^ *P*_3_^2^) + *α*_111_(*P*_1_^6^ + *P*_2_^6^ + *P*_3_^6^) + *α*_112_[*P*_1_^4^(*P*_2_^2^ + *P*_3_^2^) + *P*_2_^4^(*P*_1_^2^ + *P*_3_^2^) + *P*_3_^4^(*P*_1_^2^ + *P*_2_^2^)] + *α*_123_ *P*_1_^2^ *P*_2_^2^ *P*_3_^2^, where *α*_1_, *α*_11_, *α*_12_, *α*_111_, *α*_112_, and *α*_123_ were Landau energy coefficients [11, 51, 52]. The thermodynamic behaviors of different ferroelectric phases were determined by these coefficients. The Landau coefficients of BaTi_0.85_Zr_0.15_O_3_ under different temperatures (*T*) were set to be: *α*_1_ = 3.11 × 10^5^(*T*‐293) *C* ^−2^ *m* ^2^ *N*, *α*_11_ = 4.43 × 10^6^(*T*‐333) *C* ^−4^ *m* ^6^ *N*; *α*_12_ = −2.15 × 10^8^ *C* ^−4^ *m* ^6^ *N*, *α*_111_ = [−5.16 × 10^7^(*T*‐333) + 2.61 × 10^9^] *C* ^−6^ *m* ^10^ *N*; *α*_112_ = 2.95 × 10^9^ *C* ^−6^ *m* ^10^ *N*, *α*_123_ = 5.65 × 10^9^ *C* ^−6^ *m* ^10^ *N* according to the experimental phase transition temperatures and dielectric properties. The Landau coefficients of pure BaTiO_3_ can be found in the reference [53].

The elastic energy density can be expressed as *f*_elas_ = $\frac{1}{2}$ *C*_ijkl_(*ε*_ij_‐*ε*_ij_^0^)(*ε*_kl_‐*ε*_kl_^0^), where *C*_ijkl_ represented the elastic stiffness tensor, *ε*_ij_ donated the total strain, and *ε*_ij_^0^ was the spontaneous strain during the phase transition. The spontaneous strain correlated with the polarization through the electrostrictive coefficients *ε*_ij_^0^ = *Q*_ijkl_ *P*_k_ *P*_l_, where *Q*_ijkl_ was the electrostrictive coefficient. Specifically, *Q*_11_ = 0.1, *Q*_12_ = −0.034, *Q*_44_ = 0.029, *s*_11_ = 9.1 × 10^−12^, *s*_12_ = −3.2 × 10^−10^, *s*_44_ = 8.2 × 10^−10^. The gradient energy density can be derived as *f*_grad_ = $\frac{1}{2}$ *G*_ijkl_ *P*_i,j_ *P*_k,l_, where *G*_ijkl_ was the gradient coefficient. Notably, *G*_11_/*G*_110_ = 1.5, *G*_12_/*G*_110_ = 0, *G*_44_/*G*_110_ = 0.75, where *G*_110_ = 7.04 × 10^−11^ C^−2^m^4^N. The electrostatic energy can be formulated as *f*_elec_ =$\frac{1}{2}$(*E*·*P*), where *E* was the total electric field, expressed as *E* = *E_appl_* + *E_RF_*, with *E_appl_* representing the applied electric field and *E_RF_* denoting the local electric field caused by the random point defects. To simulate local structural heterogeneity, we introduced a certain concentration of point defects. The Fourier method was used for solving the equations to obtain a stable state of local polarization, which was then displayed as an electric domain structure at scales of 256 *dx* * 256 *dy* (2D) and 128 *dx* * 128 *dy* * 128 *dz* (3D). To simulate the hysteresis loops at different temperatures, we first simulated the 2D domain structures at different temperatures, then applied an electric field to these 2D domain structures, and finally calculated the average values of the polarization components along the *x*‐axis under different electric fields, which were used to plot the electrically induced loops at different temperatures.

### 5.13 Cell Lines and Cell Culture

The 4T1 cell line was sourced from Youze Biotechnology Co., Ltd. (Guangzhou, China), while L‐929 cells were acquired from Pricella Co., Ltd. (Wuhan, China). These cell lines were cultivated in DMEM supplemented with 10% FBS and penicillin (100 µg mL^−1^) and streptomycin (100 µg mL^−1^). Cultures were maintained in a humidified atmosphere with 5% CO_2_ at 37°C.

### 5.14 In Vitro Cell Cytotoxicity Evaluation

Cytotoxicity of BTZOX‐P3 NCs was assessed using CCK‐8 assays with and without light irradiation. 4T1 and L929 cells were seeded onto a 96‐well plate at a density of 5 × 10^3^ cells per well and incubated for 36 h. Subsequently, the media were replaced with fresh serum‐free ones containing BTZOX‐P3 (0–300 µg mL^−1^) or PBS (0.1 m, pH = 7.4) solution. After an 8 h incubation, the cells were gently washed and irradiated with or without a 1064 nm laser (1.0 W cm^−2^) for 5 mins on and 5 mins off (one cycle). Following an additional 16 h incubation, the media were removed, and the cells were washed thrice with PBS. Fresh CCK‐8 solution was added to each well, and the absorbance was measured at 450 nm after incubation.

### 5.15 Detection of Intracellular ROS

The 4T1 cell line was treated with 200 µg mL^−1^ BTZOX‐P3 for 8 h, followed by incubation with 20 µm DCFH‐DA for 30 mins. After washing three times with PBS, the cells underwent irradiation with a 1064 nm laser (1.0 W cm^−2^) for different cycles. Subsequently, the PL intensity was promptly observed by a confocal laser scanning microscopy (CLSM).

### 5.16 Live/Dead Cell Staining Assay

The 4T1 cells were initially plated at a density of 3 × 10^4^ cells per well and cultured for 24 h in a 6‐well plate. The culture medium was then replaced with 200 µg mL^−1^ BTZOX‐P3, and the cells were incubated for approximately 8 h. Following this, the cells underwent irradiation with a 1064 nm laser (1.0 W cm^−2^) for different cycles. Subsequently, the cells were cultured for an additional 4 h. After rinsing with PBS, the cells were stained using 1 µm Calcein‐AM and 1 µm PI for 30 mins. Excess dyes were washed out with PBS three times. The inverted fluorescent microscope was employed to observe the fluorescence of Calcein‐AM and PI.

### 5.17 Apoptosis and Necrosis Assay

4T1 cells were initially seeded and pre‐cultured on 6‐well plates for 24 h. The medium was substituted with 200 µg mL^−1^ BTZOX‐P3 and incubated for approximately 12 h. Following this, the cells were washed with PBS and subjected to irradiation with a 1064 nm laser (1.0 W cm^−2^) for different cycles. After the treatment, the cells were cultured for an additional 8 h. Analysis of cell apoptosis and necrosis was performed by flow cytometry using the Annexin V‐FITC Apoptosis Detection Kit.

### 5.18 Hemolysis Evaluation

The 2% red blood cells were mixed with deionized water (positive control group) or BTZOX‐P3 dissolved in PBS at different concentrations. These solutions were incubated for 4 h. Subsequently, these solutions were centrifuged for 5 min to evaluate the hemolysis condition.

### 5.19 Animal Experiments

BALB/c mice (6–8 weeks old, with an average body weight of 15–18 g) were procured from SPF Biotechnology Co., Ltd. (Beijing, China). Animal studies were approved by the Animal Ethics Committee of Fujian Normal University (20230032). All mice underwent a one‐week acclimatization period in the animal facility, maintained under pathogen‐free conditions at 25°C and 55% humidity. For the establishment of xenograft 4T1‐tumor‐bearing mouse models, 4T1 breast cancer cells (1 × 10^6^) suspended in 100 µL PBS buffer were subcutaneously injected into the right flanks of each mouse. Approximately 10 days later, tumor‐bearing BALB/c mice were randomly divided into five groups (*n* = 6): (I) control (intravenous injection with PBS), (II) BTZOX‐P3 (intravenous injection with BTZOX‐P3), (III) control+L (intravenous injection with PBS and exposure to laser for 5 min), (IV) BTZOX‐P3+L (intravenous injection with BTZOX‐P3, and exposure to laser for 5 min), (V) BTZOX‐P3+2L (intravenous injection with BTZOX‐P3, and 2L indicating exposure to laser with 5 min laser on and natural cooling, two cycles) at 8 h post‐injection on days 0 and 4. Tumor volumes were measured every other day (day 0–14) using a caliper and calculated using the formula: volume = (tumor length) × (tumor width)^2^/2. Body weights were recorded daily (day 0–14). At day 14, three mice from each group were randomly sacrificed in accordance with the approved euthanasia protocol, and the tumors were excised, fixed, and processed into sections for H&E staining, Ki‐67 immunohistochemistry, and TUNEL assays to assess histopathological changes. The remaining three mice in each group were maintained until day 18, at which point they were sacrificed, and the tumors were collected and weighed. Major organs (heart, lung, liver, spleen, and kidney) were also harvested for H&E staining to evaluate systemic toxicity. Tumor inhibition rates for each group were calculated using the formula: tumor inhibition rates = (*V*_control_—*V*_experiment_)/*V*_control_ × 100%. The survival of tumor‐bearing mice was monitored, and survival curves were constructed based on the criterion that mice were considered to have reached the experimental endpoint when the tumor volume exceeded 1500 mm^3^.

### 5.20 In Vitro and In Vivo PA Imaging

In vitro PA images and signal intensity of BTZOX‐P3 NCs at varied concentrations were obtained using a PA imaging system. For in vivo PA imaging, the BTZOX‐P3 NCs solution was injected into 4T1 tumor‐bearing mice. The PA signals and the corresponding signal intensities in tumor regions were examined using a PA imaging system at varied time points (0, 1, 4, 8, 10, 12, and 24 h).

### 5.21 Biosafety Evaluation

Blood samples were procured from mice subjected to different treatments for a comprehensive blood panel analysis. ALT, LDH, AST, CK, UA, and CR were tested for biochemical blood analysis.

### 5.22 Statistical Analysis

All data were expressed as a mean result standard deviation (S. D.). The significance of differences among groups was evaluated with a one‐way ANOVA and a student's test (^*^ *p* < 0.05, ^**^ *p* < 0.01, ^***^ *p* < 0.001, and ^****^ *p* < 0.0001).

## Author Contributions

Y.H. and H.L. designed the research, performed the experiments, and wrote the manuscript. J.Y. did the Landau free energy modeling and discussed the results. S.C did the finite element simulation. C.Z., P.W., F.S., and S.Z. performed the experiments and analyzed the data. G.D. and T.Z. performed the theoretical calculations and discussed the results. Y.D. discussed the results. X.Z., Z.Y., and W. H. supervised the research. All authors discussed the results and commented on the manuscript.

## Conflicts of Interest

The authors declare no conflicts of interest.

## Supporting information




**Supporting File**: advs74528‐sup‐0001‐SuppMat.docx.

## Data Availability

The data that support the findings of this study are available from the corresponding author upon reasonable request.
