# Supplementary material for: Boosting Photo‐Pyroelectric Effect via Tunable Polarization and Interfacial Defect Engineering
Source: Adv Sci (Weinh). 2026 Feb 21;13(25):e19280. doi: 10.1002/advs.202519280 (PMC13137801; doi:10.1002/advs.202519280)
Supplement: Supplementary file 1 — Supporting File: advs74528‐sup‐0001‐SuppMat.docx. [file ADVS-13-e19280-s001.docx]

**Supporting information**

**Boosting** **Photo-Pyroelectric Effect via** **Tunable Polarization and** **Interfacial Defect Engineering**

*Yanli Huang^1,^* *^8^, Haifen Luo^1,^* *^8^, Jie Yin^3,^* *^8^, Shuai Cao^4^, Gaolei Dong^5^, Zhi Tan^6^,Chunlin Zhao^5^, Peiye Wen^1^, Fengwei Sun^1^, Shan Zhang^1^, Xianzeng Zhang^1, *^, Yunlu Dai^7^, Zhen Yang^1, *^,* *Wei Huang ^2, *^*

**
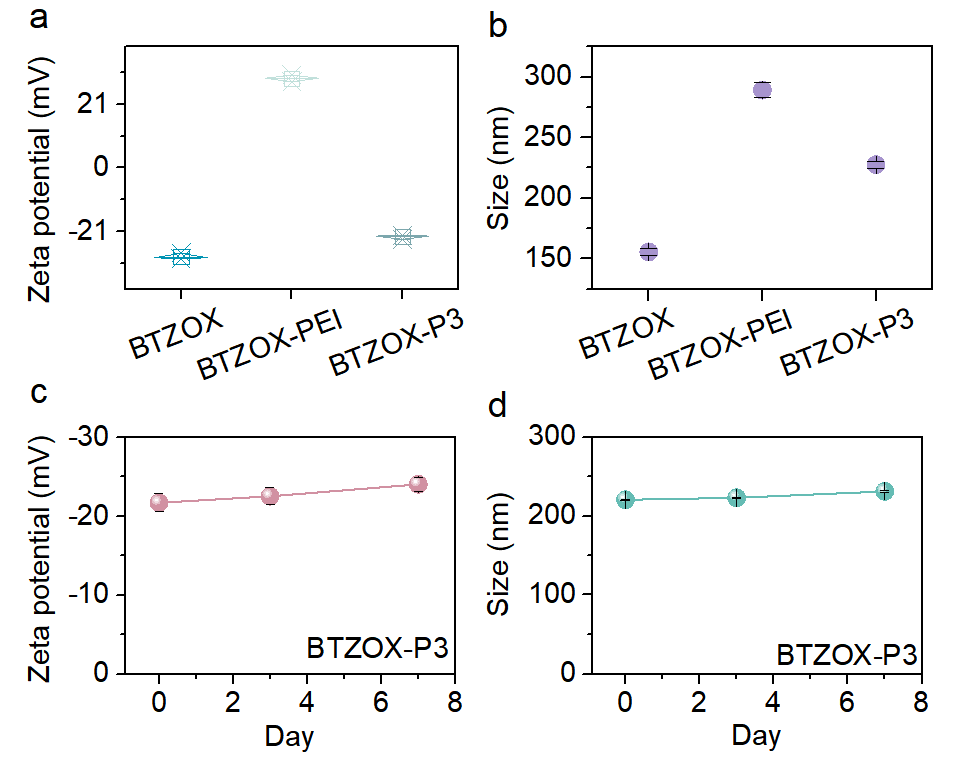
**

**Figure S1** **a** Zeta potential of BTZOX, BTZOX-PEI, and BTZOX-P3. **b** Hydrodynamic size distribution of BTZOX-P3. Time-dependent **c** Zeta potential and **d** hydrodynamic size of BTZOX-P3 in PBS over 7 days.

**
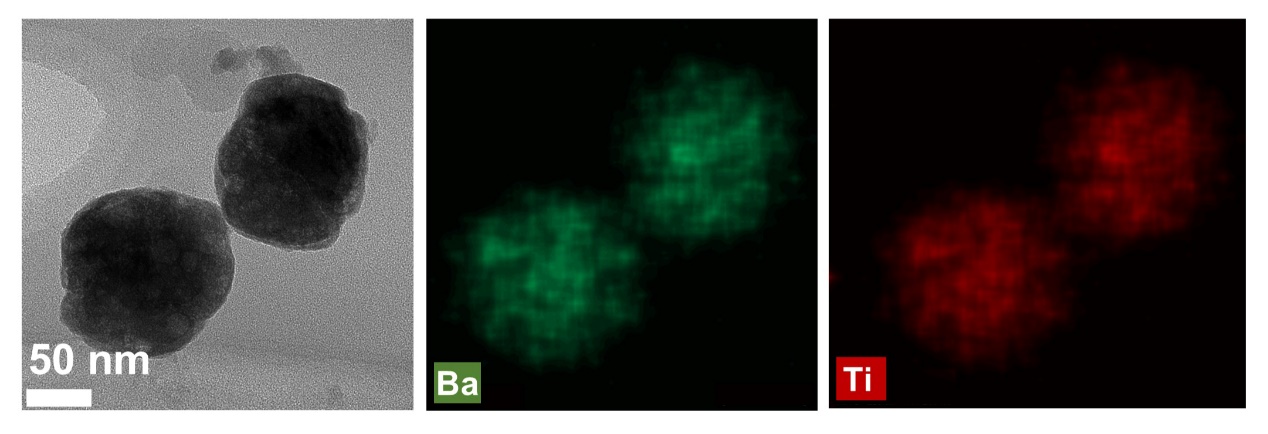
**

**Figure S2** Element mapping of BTZOX-P3 NCs. Similar TEM images were obtained for three times experiments.


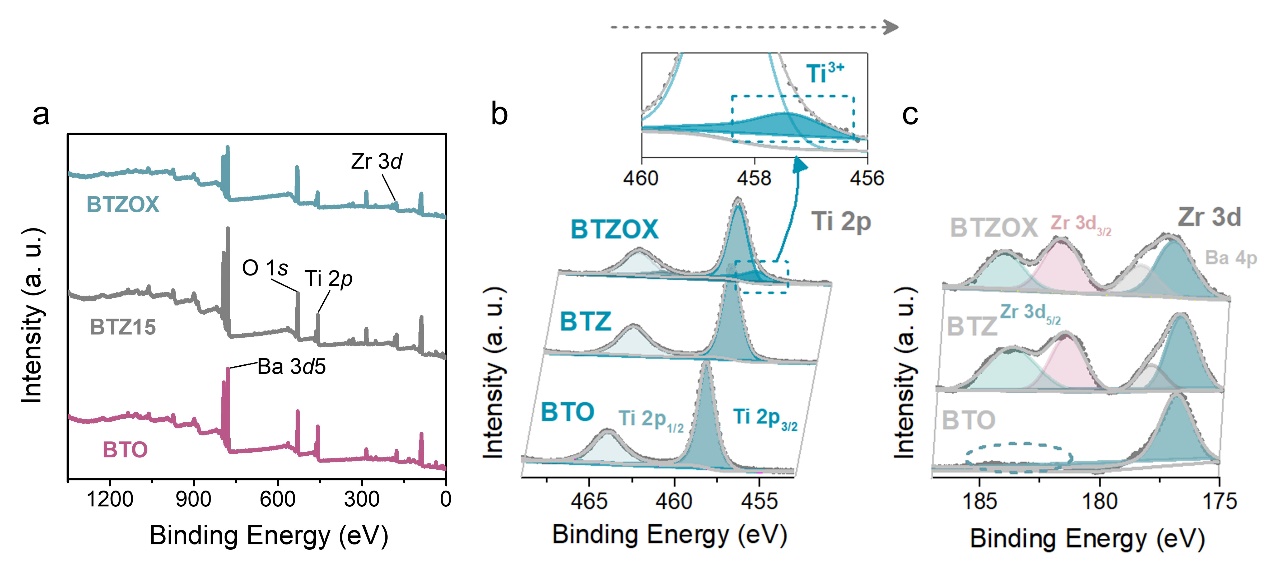


**Figure S3 a** Survey XPS spectra of BTO, BTZ, and BTZOX. High-resolution **b** Ti 2*p* and **c** Zr 3*d* spectra of BTO, BTZ, and BTZOX.


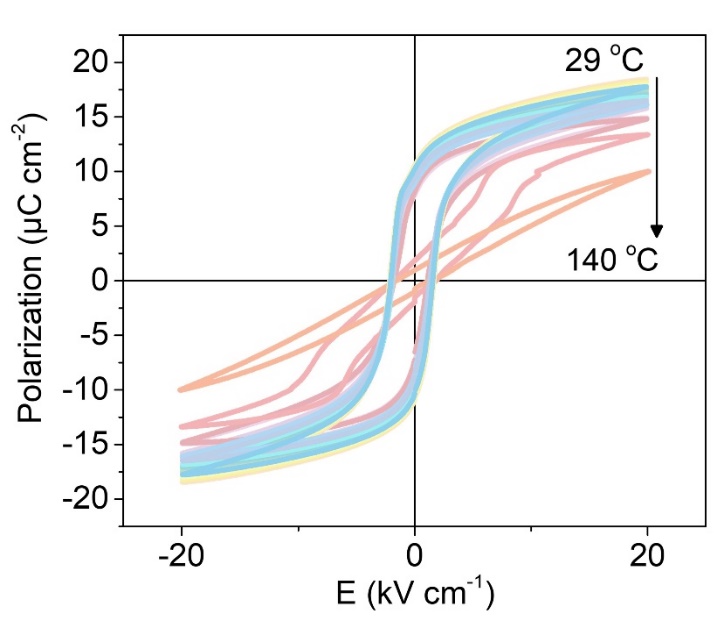


**Figure S4** *P-E* loops of BTO measured at different temperatures.


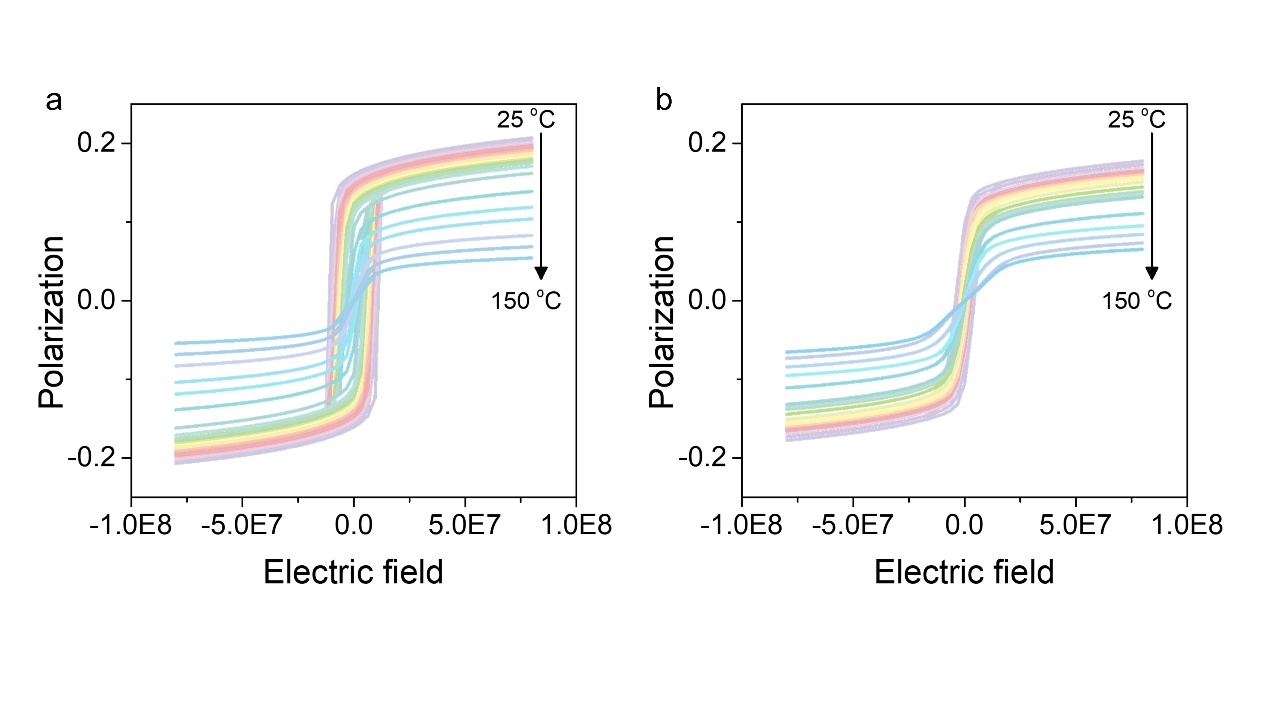


**Figure S5** *P-E* loops of **a** BTO and **b** BTZ at different temperatures by phase field simulation.


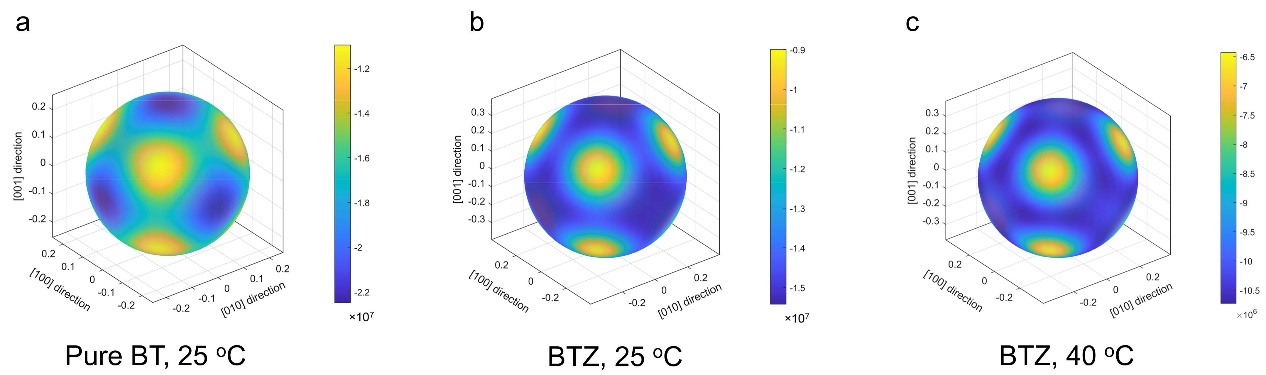


**Figure S6** Free-energy proﬁles for **a** pure BT (25 ^o^C), **b** BTZ (25 ^o^C), and **c** BTZ (40 ^o^C).


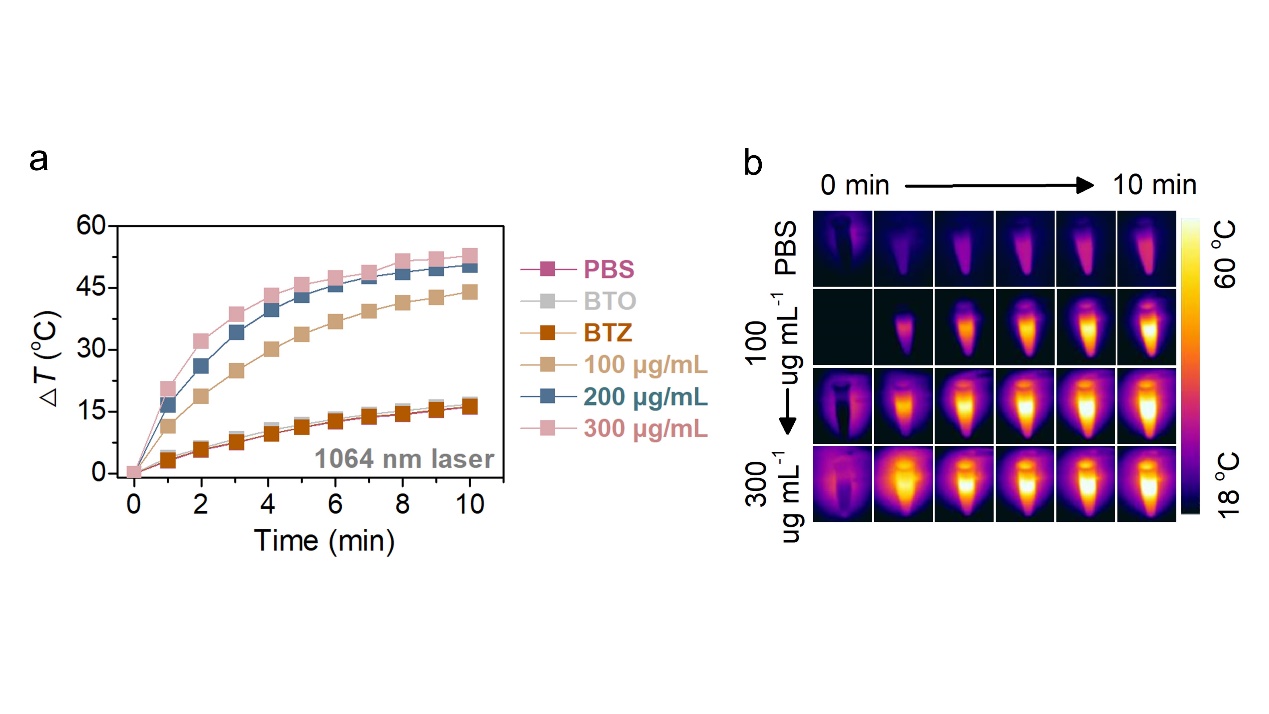


**Figure S****7** **a** Photothermal heating curves of BTZOX-P3 dispersion solution (0-300 ug mL^-1^ in 0.01 M PBS), BTO (200 ug mL^-1^ in 0.01 M PBS), BTZ (200 ug mL^-1^ in 0.01 M PBS), and PBS (0.01 M) by 1064 nm laser irradiation (1.0 W/cm^2^). **b** The infrared thermal images of BTZOX-P3 dispersion at different concentration by 1064 nm laser irradiation.


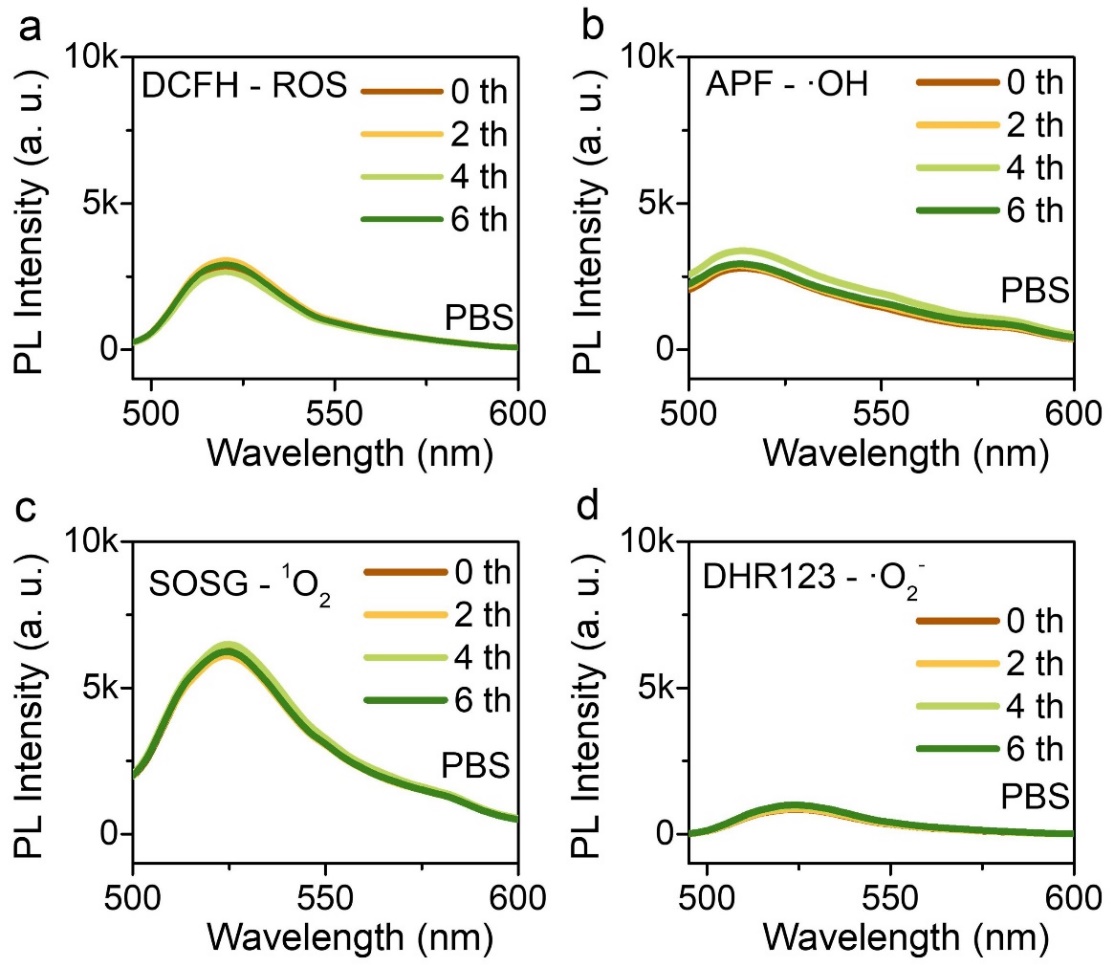


**Figure S8** The PL intensity of **a** DCFH, **b** APF, **c** SOSG, and **d** DHR123 solution after several NIR-induced heating/cooling cycles (1.0 W cm^-2^).


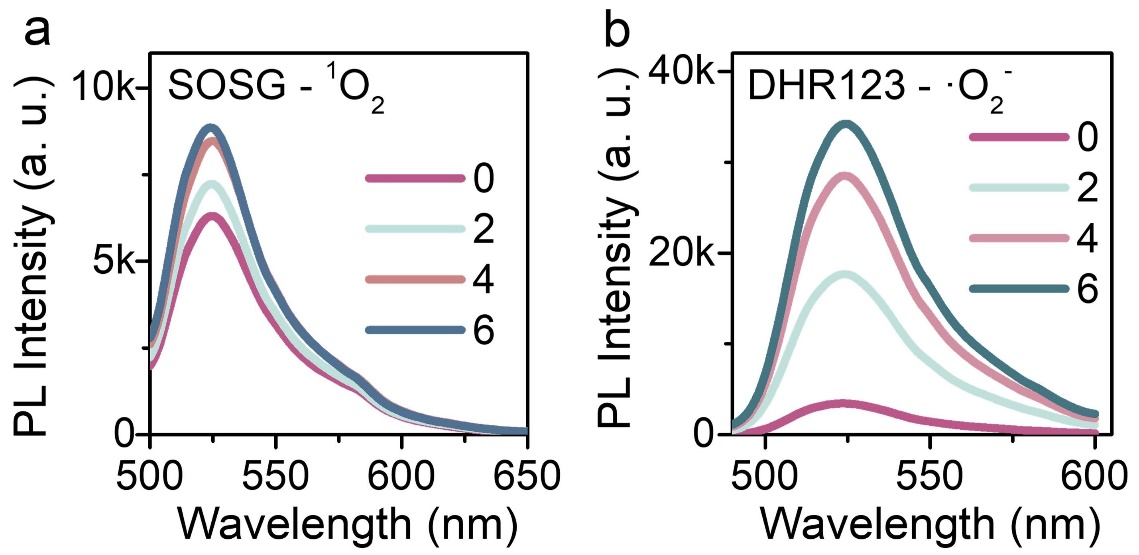


**Figure S9** The PL intensity of **a** SOSG and **b** DHR123 mixed with BTZOX-P3 nanocatalyst (200 ug mL^-1^) after several NIR-induced heating/cooling cycles (1.0 W cm^-2^).


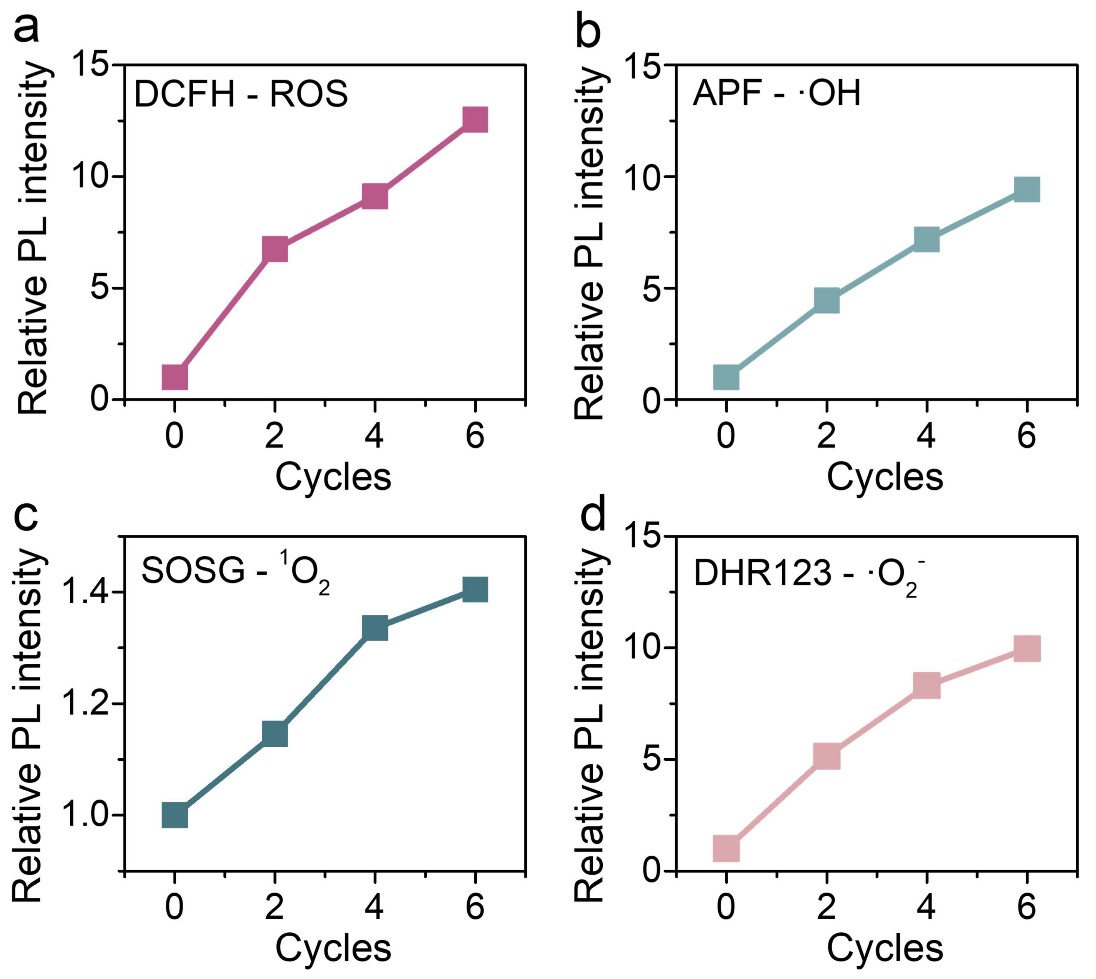


**Figure S10** Comparison of the fluorescence changes of BTZOX-P3 relative to the blank control group. **a** DCFH, **b** APF, **c** SOSG, and **d** DHR123 by 1064 nm laser irradiation (1.0 W cm^-2^) for different cycles.


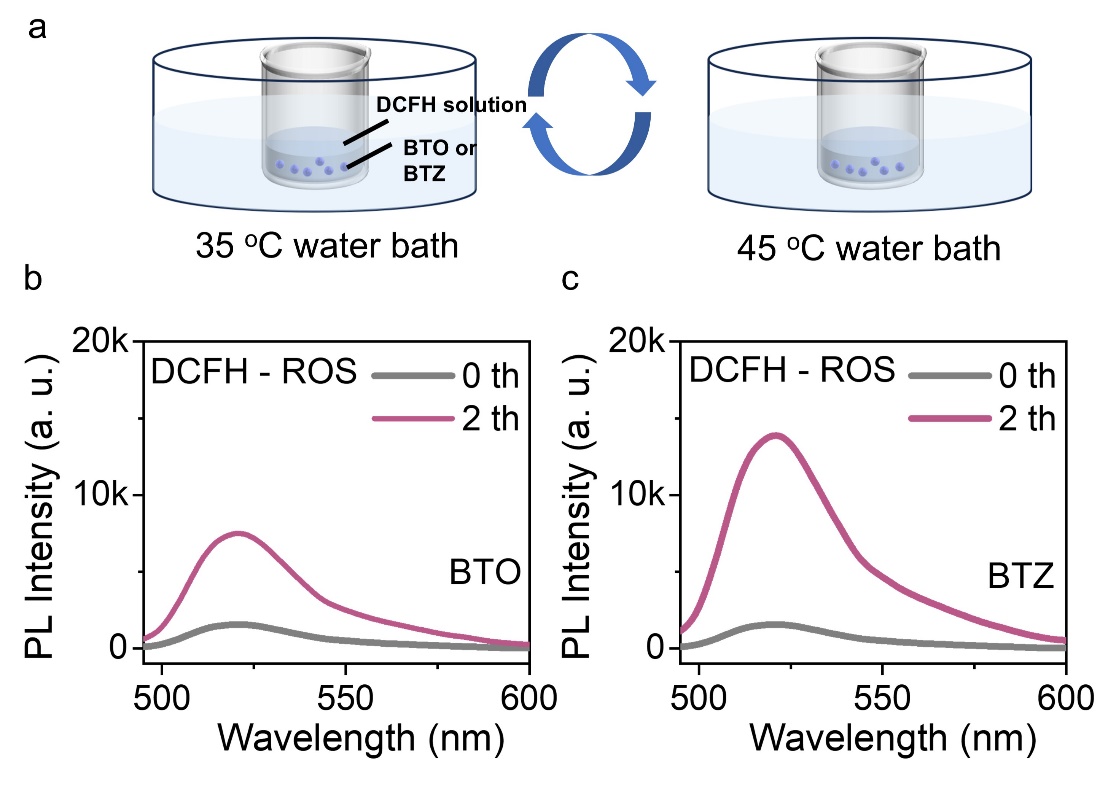


**Figure S11** **a** The schematic diagram for pyroelectric catalytic process. PL intensity of DCFH solution using **b** BTO and **c** BTZ (200 ug mL^-1^) as pyroelectric catalyst for two cycles (35 to 45 °C).


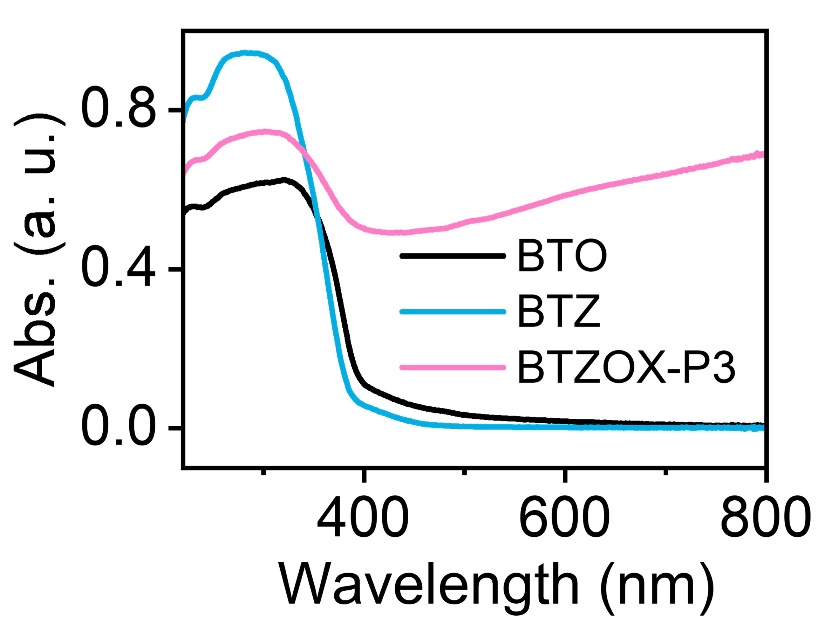


**Figure S12** UV–vis diffuse reflectance spectra of BTO, BTZ, and BTZOX-P3.


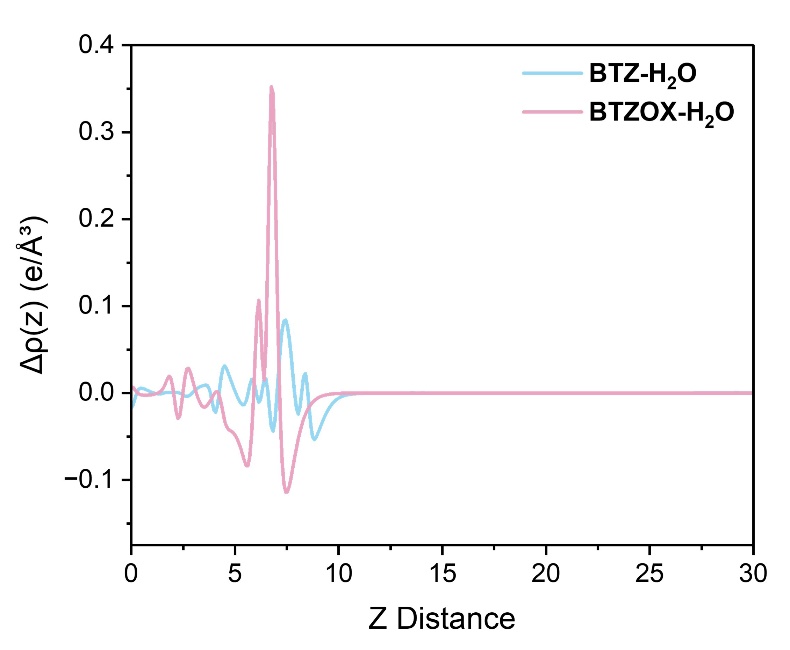


**Figure S13** The planar-averaged charge density difference of BTZ-H_2_O and BTZOX-H_2_O system.


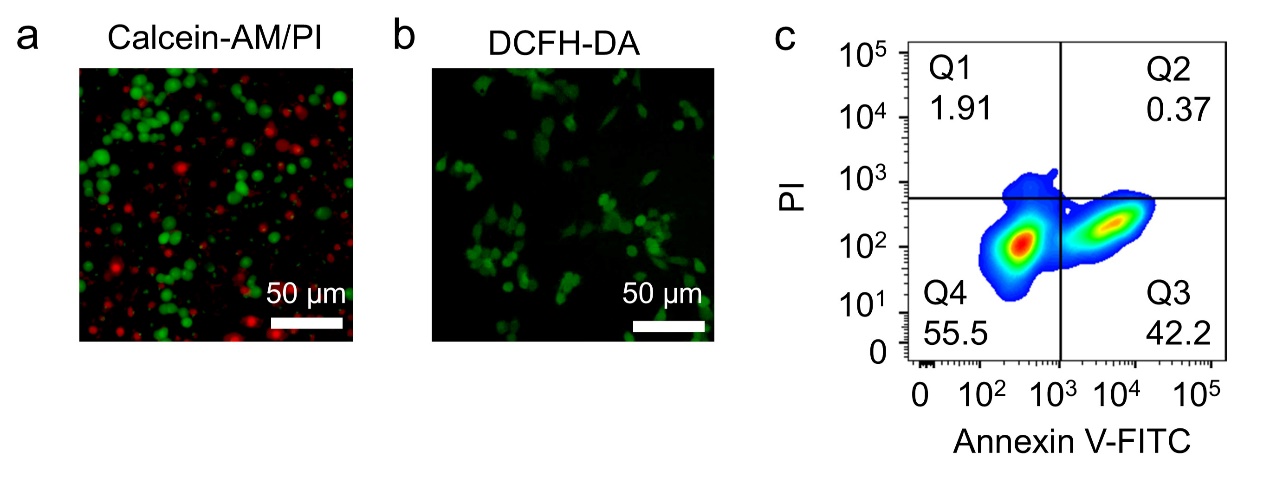


**Figure S14** **a** CLSM images of 4T1 cancer cells stained with calcein AM (green)/PI (red) after BTZOX-P3+L treatment. b DCFH-DA fluorescence images of 4T1 cancer cells with BTZOX-P3+L treatment. **c** Flow cytometry of Annexin V-FITC/PI stained 4T1 cancer cells after BTZOX-P3+L treatment.


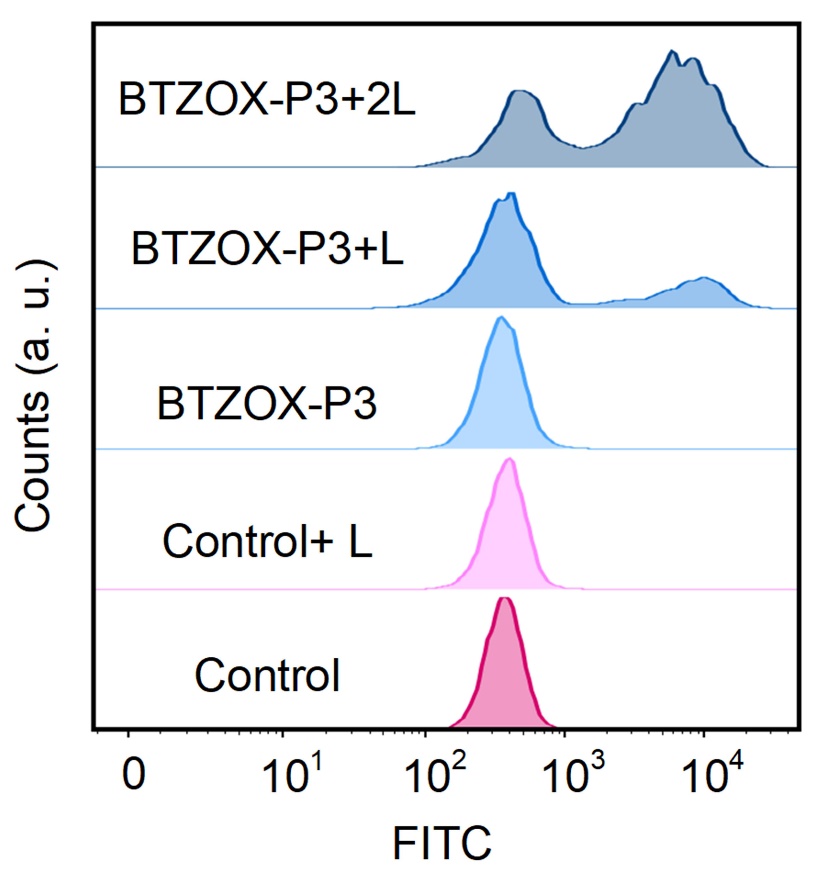


**Figure S15** Flow cytometry analysis of intracellular ROS stained by DCFH-DA.


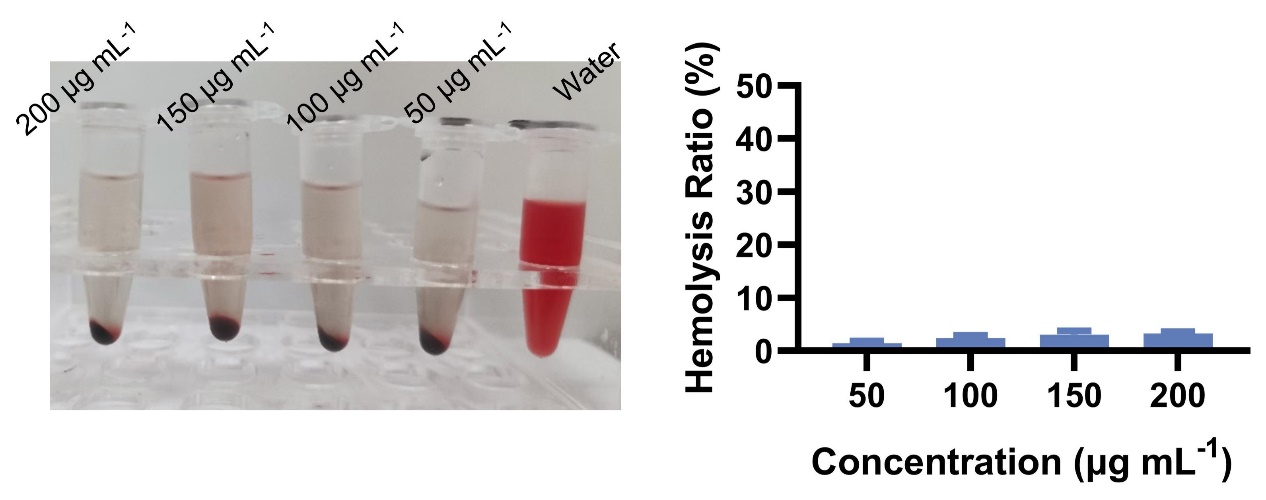


**Figure S16** Hemolysis rate of BTZOX-P3 toward red blood cells after incubation. The H_2_O acted as a positive control, n = 3 independent experiments per group.


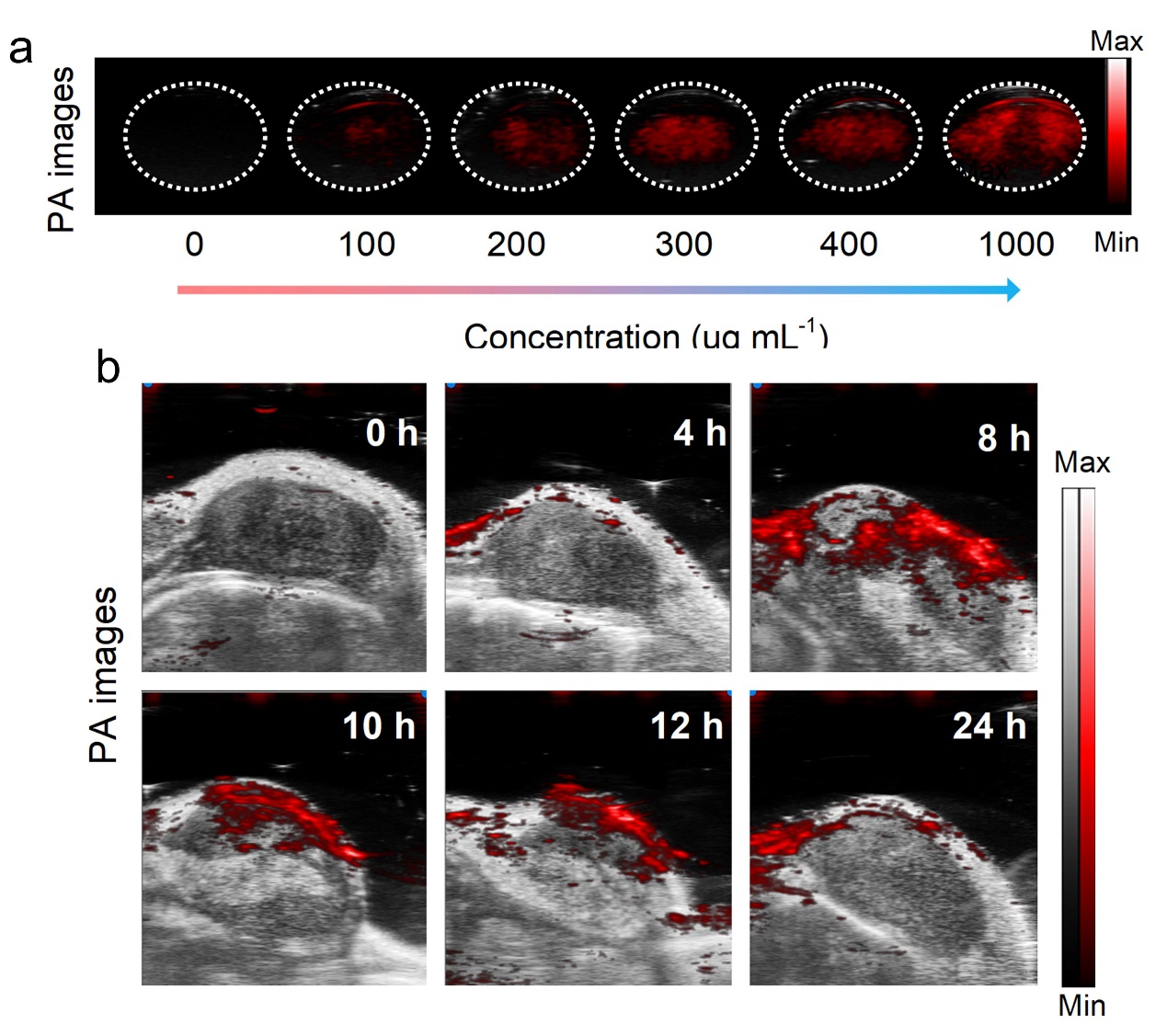


**Figure S17** **a** In vitro PA images of BTZOX-P3 suspension solution in different concentrations. **b** Time-dependent PA images within tumor sites post injection of BTZOX-P3.


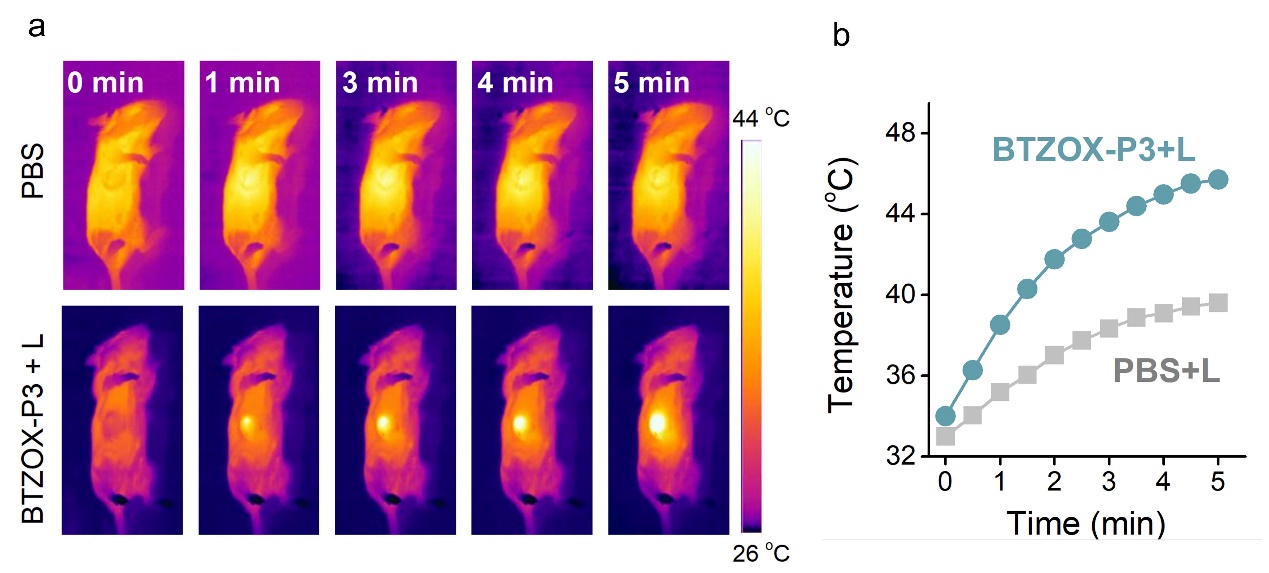


**Figure S18 a** Infrared thermal photographs of mice treated by PBS or BTZOX-P3 under 1064 nm laser exposure (1.0 W cm^-2^, 5 min). **b** The temperature of tumor region as a function of irradiation time.


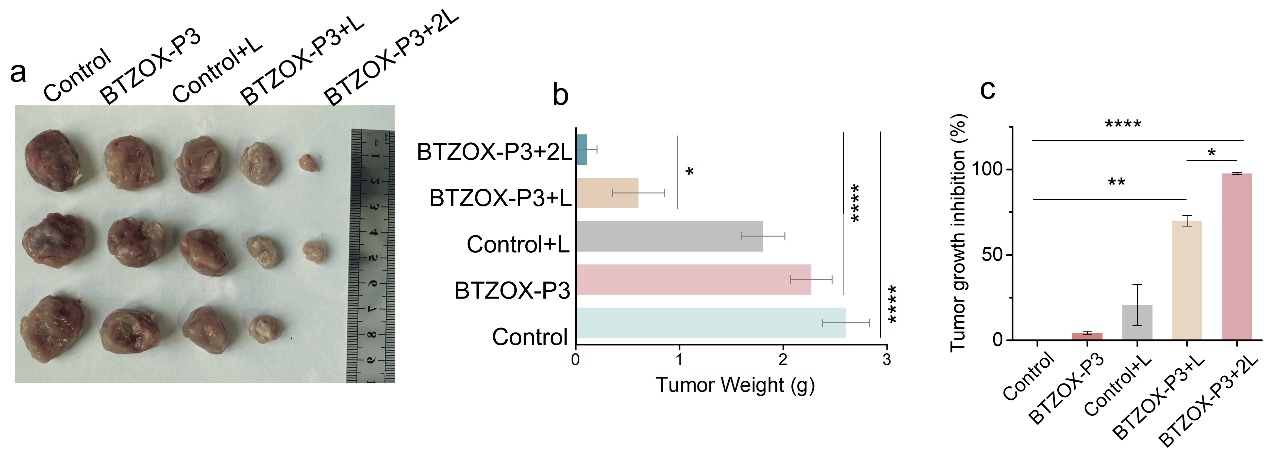


**Figure S19** **a** Photographs of excised tumors on the 18th days. **b** Weights of excised tumors on the 18th day in diverse treatment groups. n = 3 biologically independent samples, *P*-values: *< 0.05, ****< 0.0001. **c** Tumor growth inhibition after 14-day treatment. n = 6 biologically independent samples, *P*-values: *< 0.05, **< 0.01, ****< 0.0001.


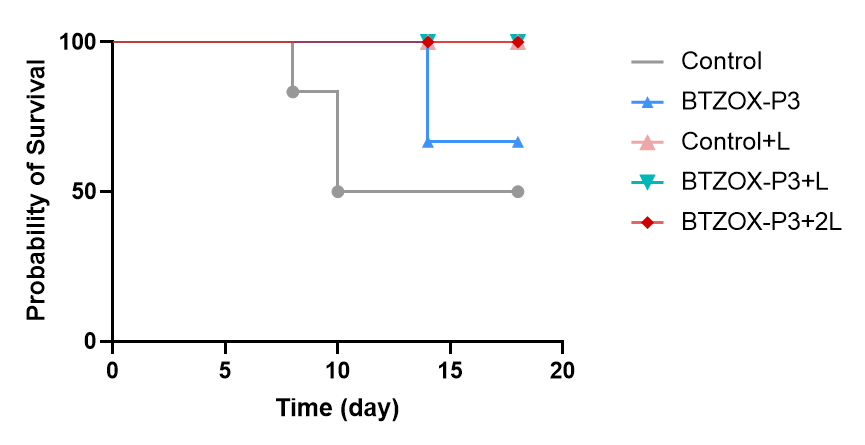


**Figure S20** The survival curves of mice after the indicated treatments, n = 6 biologically independent samples.


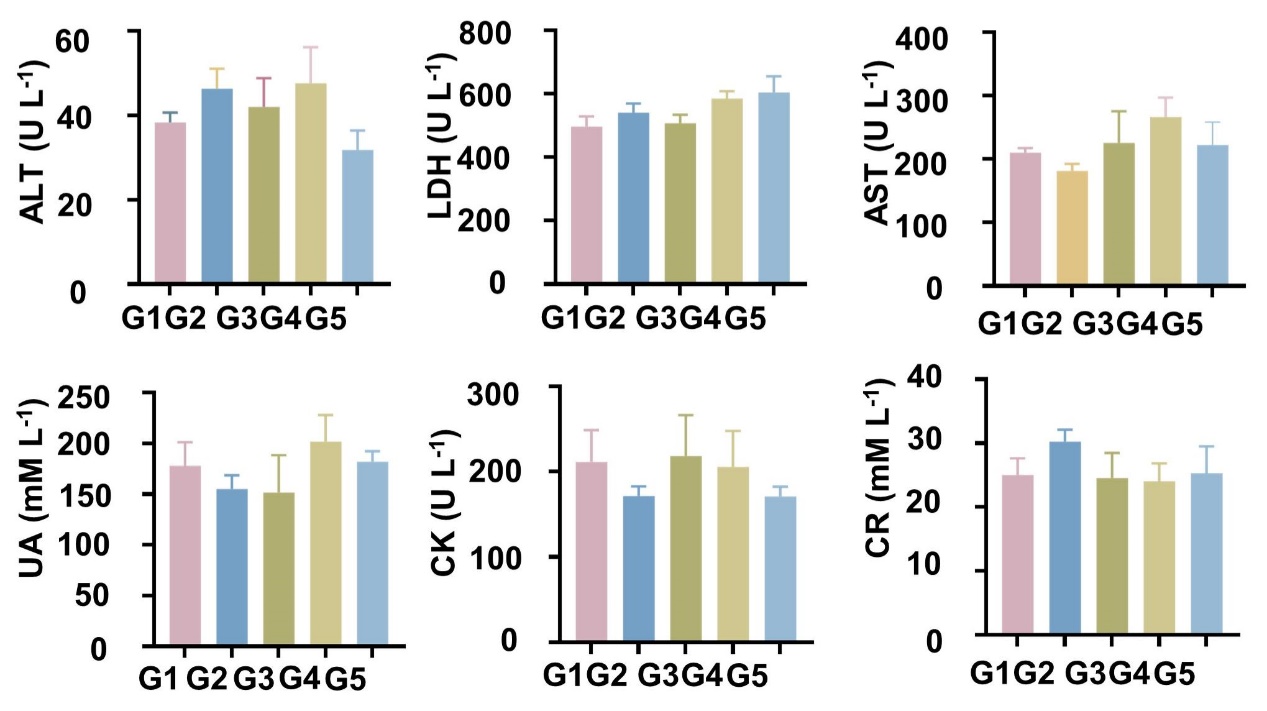


**Figure S21** Complete blood count of mice post-injection of BTZOX-P3. Mice injected with saline was used as control. G1: Control, G2: BTZOX-P3, G3: Control+L, G4:BTZOX-P3+L, G5: BTZOX-P3+2L.


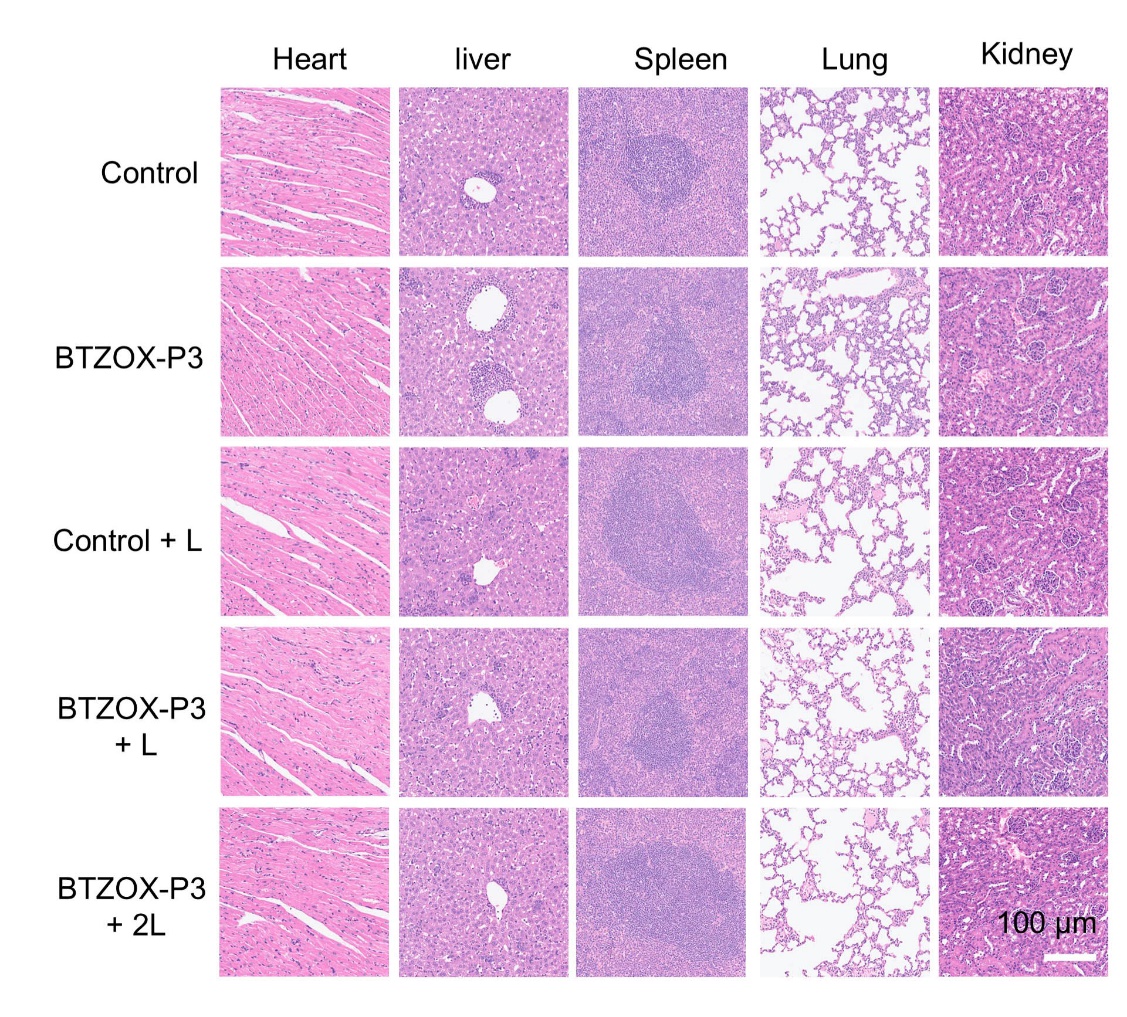


**Figure S22** Histological analysis of major organs by H&E staining after different treatments.


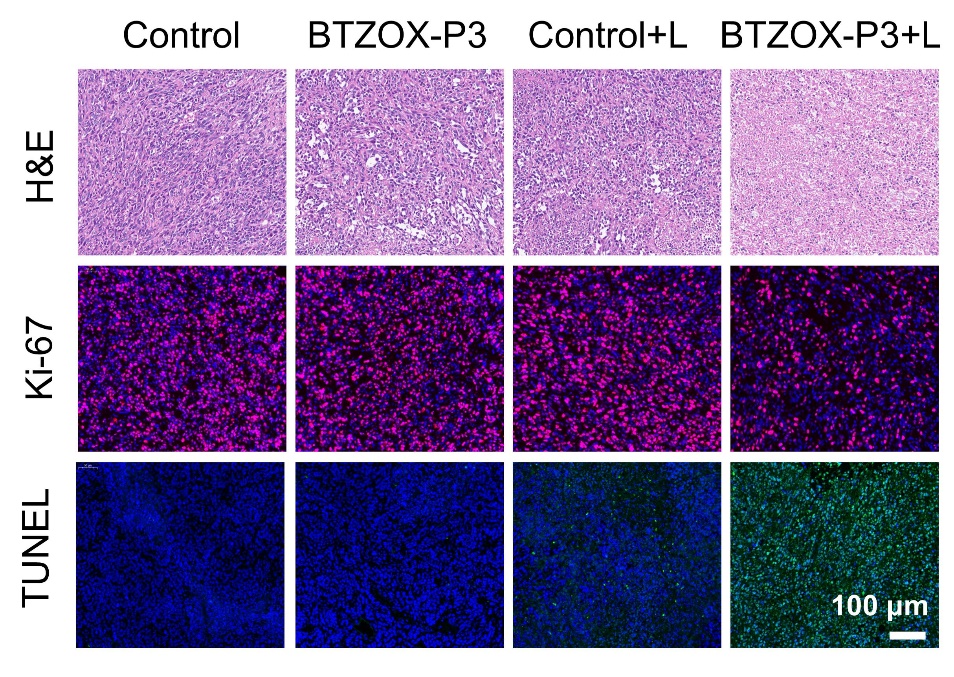


**Figure S23** H&E, Ki-67, and TUNEL staining of tumor tissues in diverse treatment groups.


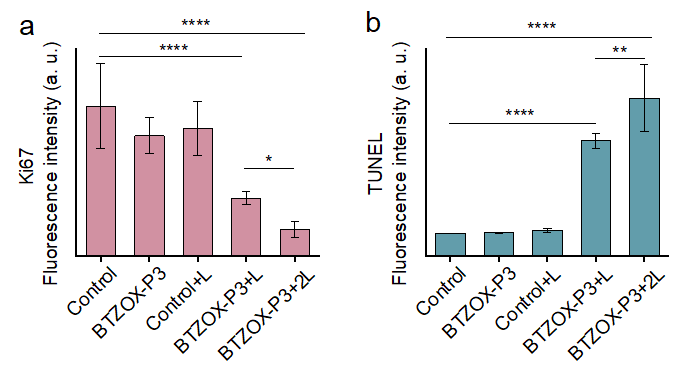


**Figure S24** The FL intensity of **a** Ki67 and **b** TUNEL of tumor tissues in diverse treatment groups. n = 3 biologically independent samples. *P*-values: *< 0.05, **< 0.01, ****< 0.0001.

**Table S1** Parameters of lattice by X-ray powder diffraction and the Rietveld refinement for BTZOX NPs at room temperature.

| Samples | Cell Parameters | | | | | | | Content (%) | *R*_w_ (%) | *Sig* |
| --- | --- | --- | --- | --- | --- | --- | --- | --- | --- | --- |
|  | a (Å) | | b (Å) | c (Å) | | *α=β=γ* (°) | |  |  |  |
| BTZOX | 4.06075 | 4.06075 | | | 4.06075 | | 89.64391 | 13 (*R3m*) | 2.72 | 1.28 |
|  | 4.02065 | 5.70043 | | | 5.72720 | | 90 | 37 (*Amm2*) |  |  |
|  | 4.01794 | 4.01794 | | | 4.03089 | | 90 | 50 (*P*4*mm*) |  |  |
